# A Mini Review on the Development of Conjugated Polymers: Steps towards the Commercialization of Organic Solar Cells

**DOI:** 10.3390/polym15010164

**Published:** 2022-12-29

**Authors:** Ahmed G. S. Al-Azzawi, Shujahadeen B. Aziz, Elham M. A. Dannoun, Ahmed Iraqi, Muaffaq M. Nofal, Ary R. Murad, Ahang M. Hussein

**Affiliations:** 1 Department of Chemistry, University of Sheffield, Sheffield S3 7HF, UK; 2 Department of Chemistry, College of Education for Pure Science, University of Mosul, Mosul 00964, Iraq; 3 Hameed Majid Advanced Polymeric Materials Research Lab., Department of Physics, College of Science, University of Sulaimani, Qlyasan Street, Sulaimani 46001, Iraq; 4 The Development Center for Research and Training (DCRT), University of Human Development, Sulaimani 46001, Iraq; 5 Associate Chair of the Department of Mathematics and Science, Woman Campus, Prince Sultan University, P.O. Box 66833, Riyadh 11586, Saudi Arabia; 6 Department of Mathematics and Science, Prince Sultan University, P.O. Box 66833, Riyadh 11586, Saudi Arabia; 7 Department of Pharmaceutical Chemistry, College of Medical and Applied Sciences, Charmo University, Chamchamal, Sulaimani 46023, Iraq

**Keywords:** conjugated polymers, bulk heterojunction (BHJ) device, organic solar cells, Suzuki coupling reaction, Sonogashira coupling reaction, benzothiadiazole (BT), naphthothiadiazole (NT)

## Abstract

This review article covers the synthesis and design of conjugated polymers for carefully adjusting energy levels and energy band gap (EBG) to achieve the desired photovoltaic performance. The formation of bonds and the delocalization of electrons over conjugated chains are both explained by the molecular orbital theory (MOT). The intrinsic characteristics that classify conjugated polymers as semiconducting materials come from the EBG of organic molecules. A quinoid mesomeric structure (D-A ↔ D^+^ = A^−^) forms across the major backbones of the polymer as a result of alternating donor–acceptor segments contributing to the pull–push driving force between neighboring units, resulting in a smaller optical EBG. Furthermore, one of the most crucial factors in achieving excellent performance of the polymer is improving the morphology of the active layer. In order to improve exciton diffusion, dissociation, and charge transport, the nanoscale morphology ensures nanometer phase separation between donor and acceptor components in the active layer. It was demonstrated that because of the exciton’s short lifetime, only small diffusion distances (10–20 nm) are needed for all photo-generated excitons to reach the interfacial region where they can separate into free charge carriers. There is a comprehensive explanation of the architecture of organic solar cells using single layer, bilayer, and bulk heterojunction (BHJ) devices. The short circuit current density (*J_sc_*), open circuit voltage (*V_oc_*), and fill factor (FF) all have a significant impact on the performance of organic solar cells (OSCs). Since the BHJ concept was first proposed, significant advancement and quick configuration development of these devices have been accomplished. Due to their ability to combine great optical and electronic properties with strong thermal and chemical stability, conjugated polymers are unique semiconducting materials that are used in a wide range of applications. According to the fundamental operating theories of OSCs, unlike inorganic semiconductors such as silicon solar cells, organic photovoltaic devices are unable to produce free carrier charges (holes and electrons). To overcome the Coulombic attraction and separate the excitons into free charges in the interfacial region, organic semiconductors require an additional thermodynamic driving force. From the molecular engineering of conjugated polymers, it was discovered that the most crucial obstacles to achieving the most desirable properties are the design and synthesis of conjugated polymers toward optimal p-type materials. Along with plastic solar cells (PSCs), these materials have extended to a number of different applications such as light-emitting diodes (LEDs) and field-effect transistors (FETs). Additionally, the topics of fluorene and carbazole as donor units in conjugated polymers are covered. The Stille, Suzuki, and Sonogashira coupling reactions widely used to synthesize alternating D–A copolymers are also presented. Moreover, conjugated polymers based on anthracene that can be used in solar cells are covered.

## 1. Background

The technology of organic semiconductors has developed and progressed remarkably since the early work on polyacetylene (PA) which emerged in 1976 as the simplest conjugated polymer. The conducting ability of the polymers was discovered by Shirakawa and his talented group when they doped PA with iodine [1,2,3,4]. This discovery opened up a new field of research on the electronic technology that is considered the first generation semiconducting polymers [5]. The second generation of conjugated polymers was introduced as soluble and stable polymers such as poly(3-alkylthiophenes) (PT) and poly(*p-*phenylene vinylene) (PPV) due to alkyl chains and heteroatoms over the polymer chains [2,5,6]. The third generation of organic semiconductors has more complex structures with more atoms in the main chains of the conjugated polymers such as donor–acceptor (D–A) alternating copolymers along the main chains. These materials have emerged in the past few years as stable semiconducting materials. The main target of synthesis of D–A conjugated copolymers is to attain high-efficiency materials due to alternating electron-deficient and electron-rich units along the backbones of conjugated polymers, which will be discussed later [5,7].

**Figure 1:**
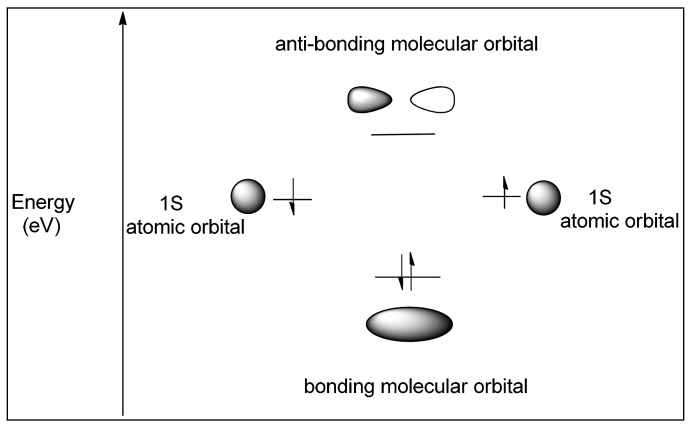
Molecular orbital energy levels diagram demonstrating the formation of σ-bonding and σ^×^ -antibonding molecular orbitals.

The prototype of conjugated polymers (PA) was described by Natta et al. in 1958 and prepared using the Ziegler–Natta catalyst before the conductivity of this type of polymer was discovered [1,4,8]. Later, it was realized that the main chains of conjugated PA might have various interesting properties, especially optical, electrical, and magnetic properties [1,9]. To explain the unique optoelectronic properties and conductivity, it is necessary to demonstrate the electronic structure of the repeat units of the polymer. PA is commonly considered as a model system for describing a prototype conducting polymeric material. Compared with saturated molecules, σ-bonded hydrocarbon chains possess large EBG and are considered insulating materials, such as polyethylene which has an optical EBG of around 8 eV [10,11,12]. With respect to the conjugated polymer, it is an unsaturated molecule that consists of alternating single and double bond C–C atoms over its main chain. The main backbone of PA has three covalent σ-bonds between adjacent C atoms and H atoms due to overlapping sp^2^ hybrid orbitals in the plane geometry; these bonds hold the main structure chain of the PA together. The π-bonds in the chain of the polymer come from the unhybridized *P_z_* orbital on each C or N atom, which lies perpendicular to the plane structure. The overlaps with the *P_z_* orbital on the neighboring atom via the interaction to form π-bonds result in delocalized π-electrons over the long chains [1,13,14]. In order to understand how the optoelectronic properties of the conjugated polymer in organic photovoltaic devices are modulated, molecular orbital theory (MO) is essential to explain the bond formation and delocalized electrons over the conjugated chains [15]. Generally, the conjugated polymers have σ- and π-bonds over the main chains, and σ-bonds are formed when two atomic orbitals overlap resulting in two molecular orbitals having different energy levels. The low-energy molecular orbital is called the bonding (σ) molecular orbital, and is formed as a result of the in-phase atomic orbitals’ overlap (constructive interference) causing an increase in electron density along the bond axis. The molecular orbital of increased energy is referred to as the anti-bonding (σ^×^) orbital due to the out-of-phase atomic orbitals that interfere destructively resulting in low electron density between the two nuclei [16,17], as shown in Figure 1.

**Figure 2:**
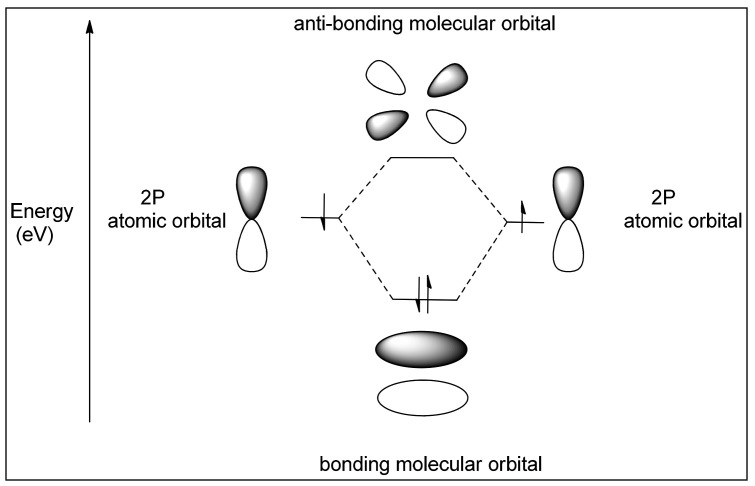
The diagram of molecular orbitals showing the formation of π- and π*-bonding orbitals.

In terms of energy, the difference energy, commonly known as EBG, is referred to as the required energy that excites an electron from the bonding orbital (the valence band) to the anti-bonding orbital (the conduction band). As mentioned before, the hydrocarbon molecules containing only σ-bonds are considered insulators with relatively large EBG resulting in no electrons accommodating the anti-bonding molecular orbital, and thus no charge carriers migrating through the alkyl-chain structure [16]. π-bonds (Figure 2) are formed through the overlap of atomic *P_z_* orbitals that are parallel to other carbon atoms and perpendicular to the atom axis. This attraction between atomic orbitals forms a π-molecular orbital which is spread over and below the atomic orbitals with different energy levels. The molecular orbital of reduced energy is termed the bonding (π) orbital (Highest Occupied Molecular Orbital HOMO), whilst the molecular orbital of increased energy is referred to as the anti-bonding (π*) orbital (Lowest Unoccupied Molecular Orbital, LUMO) [15].

**Figure 3:**
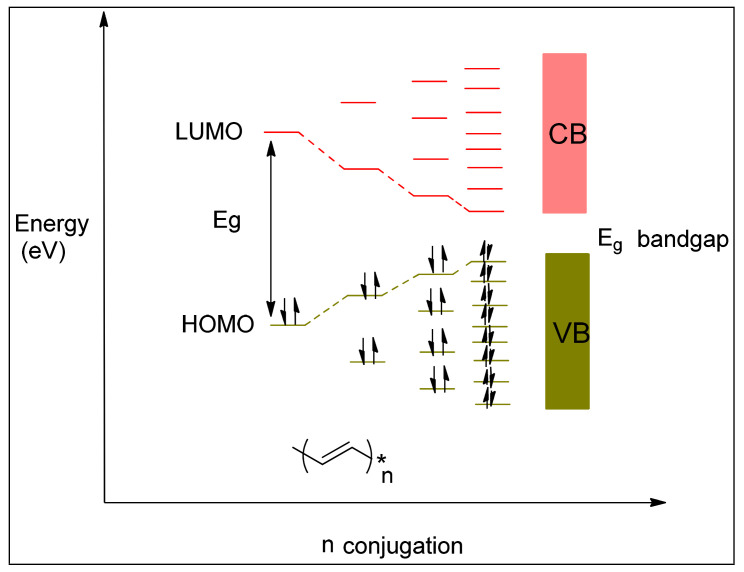
Energetic diagram profile showing the effect of increasing the number of conjugated π-bonds on the magnitude of the EBG of the conjugated polymer.

The differences in these energy levels generate π-bands (commonly known as EBG). The resulting π-bond is shorter than a σ-bond due to the effect of pulling carbon atoms closer to each other, resulting in narrow EBG [15]. The EBG of organic molecules are the origin of intrinsic properties that characterize conjugated polymers as semiconducting materials. In addition, the HOMO–LUMO energy gap is determined by the number of sub-bands in low and high energy levels that are dependent on the number of carbon atoms in the repeat unit of the polyene [1,13,14]. The energy gap (Figure 3) between HOMO and LUMO is decreased as a sequence of extended conjugation in the main chain, resulting in more thermodynamically stable molecules when compared to the same number of saturated hydrocarbon chains [15]. Overall, the EBG of conjugated polymers containing alternating single and double bonds is smaller than the EBG of non-conjugated materials and is in the range 1 to 4 eV [10]. The HOMO–LUMO EBG of organic molecules determines the absorption profile; thus, this EBG illustrates a relation between the absorption profile and harvesting ability of the conjugated polymer in organic devices with increasing numbers of repeating units in the π-system [14,18].

**Figure 4:**
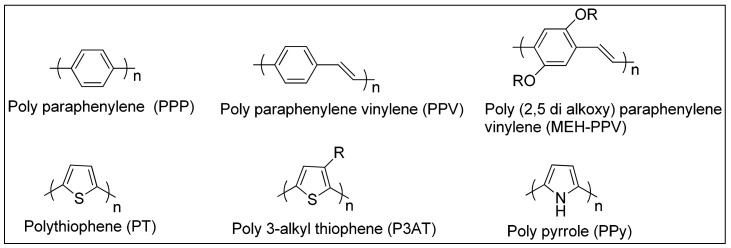
Molecular structures of some important heterocyclic polymers (second generation).

Generally, this is not the whole picture for conducting conjugated polymers. Most of the conjugated polymers are disordered structures, which are termed amorphous materials. Weak interchain interactions of these molecules are one of the defects of polymer chains; this weakness [19] causes energy barriers for π-electron delocalization and reduces the chain packing [1]. In addition, disordered structures result in localization of the electron–hole pairs that are more strongly bound by Coulomb interactions [1,10,20]. Polymers such as PA, which has a relatively enlarged π-electron system in its backbone, are insulators (not conductive) in their native state. However, this state can be transformed into a conductive state with a doping processes [1] (oxidative dopant or reductive dopant) in order to exhibit metallic and conducting properties for the polymer. This process converts the insulating neutral polymer into a (cation or anion) complex that is the reduced or oxidized form of the conducting material [1,21,22,23]. Although PA is one of the most-conducting polymers studied today, its instability in air and tedious processability are still amongst the challenges [6] that have motivated the research community to develop other stable and easily processable polymeric materials. Such materials are based on aromatic structures or heterocycles having a five-membered ring structure in the main chain. The molecular structures of this second generation of semiconducting polymers [5,6] are depicted in Figure 4. For example, polythiophene and poly(phenylene vinylene) were made soluble and processable via attaching alkyl or alkoxy side chains [5]. Nevertheless, these species do not possess ideal EBG, which negatively impacts their sunlight-harvesting ability as polymers [24].

The third generation of semiconducting polymers has emerged in the past few years as a result of developments in polymer-based photovoltaic elements. These polymers have more complex molecular structures with different atoms in the repeat units with either heterocycles or benzene rings [5]. Incorporation of alternate electron-donor–acceptor units over the polymeric backbone decreases the EBG of the polymer, resulting in an enhancement of the absorption ability of the organic semiconducting material [25]. During the last few years, remarkable progress has been achieved in this field: the efficiency of these materials in devices has exceeded 9% [26]. It is well recognized that substantial features and properties of conjugated polymers play crucial roles in the overall performance of organic photovoltaic (OPV) devices [27]. Therefore, an enormous number of conjugated polymers has been reported for application in electronic devices, leading to a wide variety of physical and mechanical properties as a consequence of the various organic syntheses of the conjugated polymers [7,28]. Alternating electron-rich and -deficient motifs in the main backbone of the polymer result in the tuning of its energy levels and its EBG [28,29]. The aim of the D–A approach is to obtain a narrow EBG and a highly ordered chain structure. Moreover, incorporation of alternate donor–acceptor motifs into the polymer chains can facilitate intermolecular interactions [25,30,31]. These interactions can enhance electron transfer in the organic cell [29]. On the other hand, the disordered structure and poor crystallinity of conjugated polymers have considerable influence on charge-carrier mobility and performance. Additionally, large EBG organic semiconductors limit the absorbing ability of devices compared to traditional crystalline silicon devices, which have an efficiency of 25%. Consequently, the performance of organic devices is greatly limited [32,33]. Nevertheless, the organic photovoltaic devices have received great attention in both research and industrial communities as a consequence of their potential advantages, such as light weight, affordable costs, and ease of manufacture to produce plastic devices for renewable energy [28,34]. Furthermore, the chemical modification and manipulation of synthetic methods of these organic materials introduce various intrinsic and promising properties that have caught the attention of the research community [32,34]. Despite great progress, organic solar cells have not yet reached the industrial market, unlike traditional inorganic solar cells [35].

## 2. Application of Conjugated Polymers

The novelty of conjugated polymers as semiconducting materials is employed in a broad spectrum of applications, as they could combine excellent optical and electronic properties as well as good thermal and chemical stability. Furthermore, these materials contribute to a wide range of applications such as electroluminescent diodes and field-effect transistors besides plastic solar cells, which were discussed above [29]. All these applications have produced several products that are commercially available in global markets [36,37]. Many conjugated polymers have been intensively investigated and applied for these applications [29].

### 2.1. Light-Emitting Diodes

**Figure 5:**
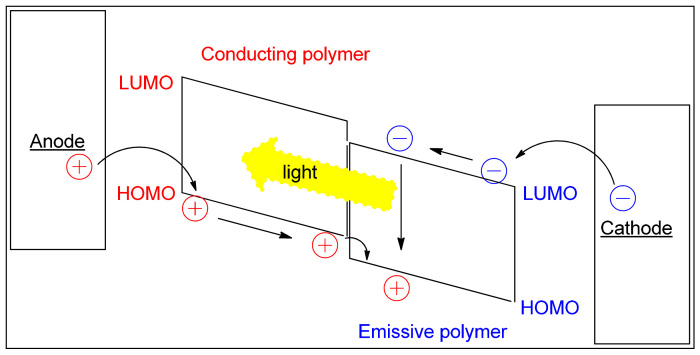
Essential parts of an OLED.

Tremendous research efforts after discovering electroluminescence (EL) in conjugated polymers have led to wide innovations and developments in organic light-emitting diodes (OLEDs). The potential for cheap, easy manufacturing and incorporation into devices of conjugated polymers has attracted considerable interest for their application in light-emitting devices [38,39]. It is important to report that EL products such as digital cameras and mobile phones are some of the kinds of flat display panels that are already commercialized. However, this technology still faces big challenges with regards to the upgrade of performance and solidity of OLEDs for lighting and displays. The basic principle of the mechanism of OLEDs is the opposite principle to that in organic solar cells (OSCs). Basically, the organic emissive and conductive layers are sandwiched between a transparent anode (high-work function electrode) and cathode (low-work function electrode) [40]. Injection of holes and electrons from opposite sides of the device and migration of these into the films and their recombination generates excitons (Figure 5), which can then decay and radiate visible light [40,41].

The wavelength of the light emission depends on the EBG of the conjugated polymer used in this application, while the intensity and brightness of the emitted light are proportional to the injected current in the device. This is the basic principle of the functioning of this technology [40]. It is hard to implement highly efficient OLEDs with the single emissive layer configuration, as it gives low efficiency and brightness. The performance of OLEDs can be improved by using two or more different materials that help to promote the required function of efficient light emission and create a good hole–electron junction in an organic emitter layer [39,40].

### 2.2. Field-Effect Transistors (FET)

**Figure 6:**
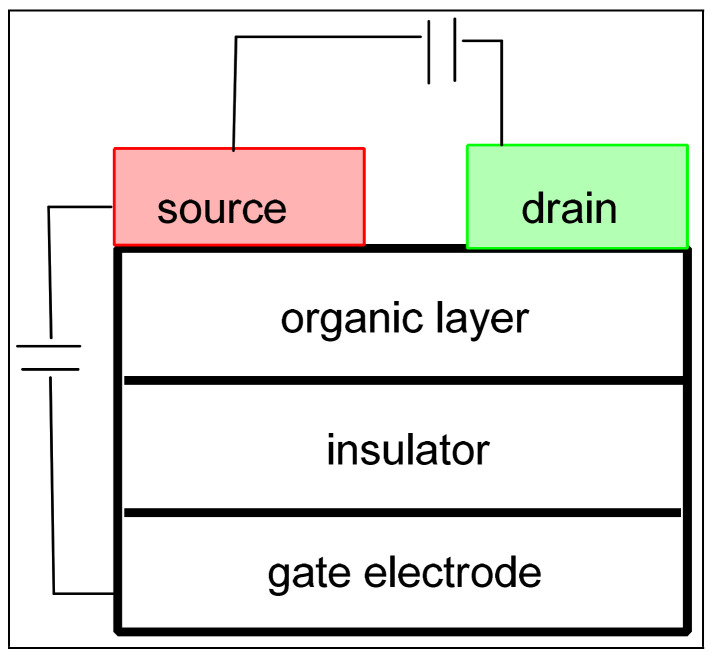
Schematic diagram of the device configuration of an OFET.

Organic semiconductors had been used in field-effect transistors in 1970. From that time, tremendous efforts were devoted in developing this type of transistor for electronic applications [42]. Conjugated polymers display promising properties such as low cost, flexibility, and high mobility that make them good candidates for use in transistors. Hence, organic field-effect transistors (OFETs) are preferred to amorphous silicon, which is considered as conventional crystalline silicon. Basically, an OFET is composed of a thin film of an organic material, as shown in Figure 6. This is applied between two ohmic contacts (source and drain) as well as the third electrode (insulted gate electrode), which is used to change the conductivity of the contacts to control the amount of current flow between ohmic contacts [43,44].

Organic semiconductors play a crucial role in determining the device’s performance. Therefore, high-carrier-mobility polymers are required in these applications. The high-carrier-mobility polymers generally have linear fused-ring structures such as benzene and thiophene molecules. There are many requirements for the polymers to be appropriate for the OFET industry; for example, they should have high charge-carrier mobility, good solubility, and be synthetically inexpensive. OFETs are the essential part in different industrial applications such as modern circuitry, single amplifiers, and electrical switches [44].

## 3. Architecture of Organic Solar Cells

In the history of organic-material-based electronic devices, a single layer sandwiched between two electrodes was the first design used for organic solar cells. This single-layer design has been replaced by a bilayer junction in order to improve the performance of the device. The major breakthrough and rapid development of configuration of these devices have been achieved since the bulk heterojunction (BHJ) concept was introduced. This discovery led to an efficient charge transport in conjugated polymers. Further development of the BHJ design was accomplished via enhancement of the morphology of the active layer to secure efficient exciton (electron–hole pair) dissociation and charge mobility. Although OPVs have been rapidly improving in terms of their efficiency, there are still challenges that are not fully resolved such as further improvements in device efficiencies, low lifetimes of devices, stability, and large-scale production [45].

### 3.1. Single-Layer Device

**Figure 7:**
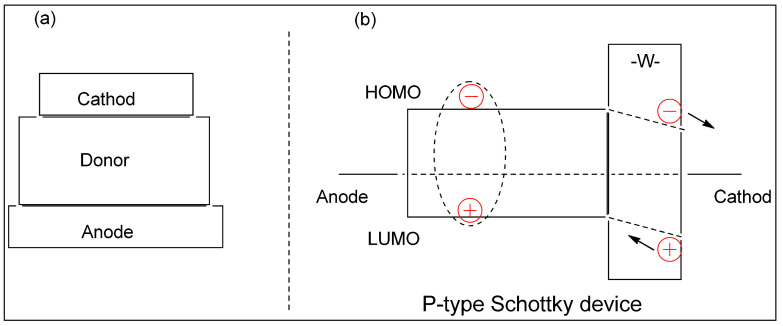
(**a**) Schematic of a single organic device. (**b**) In a p-type Schottky contact at the metal cathode, photogenerated excitons are dissociated in a thin depletion region W.

The earliest organic solar cell (Figure 7) was based on a single photoconductive material sandwiched between two metals with different work function electrodes. In an organic single device, a photovoltaic effect can arise due to symmetry of the organic device [32,46], which results in the formation of a Schottkybbarrier. This barrier is formed between the p-type (hole transporter) organic component and the low-work-function metal as cathode (electron transporter) [32,47]. Exciton dissociation occurs at the cathode interface but excitons are not completely free and tightly bonded. Therefore, only photo-generated excitons that reach the dissociation interface can split and generate free charge carriers. Consequently, as a result of light absorption by the active layer, it is important to highlight that the free-charge concentration into the photoactive layer is low. The first fabricated single-layer device exhibited low efficiencies in the range of 10^−3^–10^−2^% [32], which can be attributed to a loss mechanism caused by short exciton-diffusion lengths in the construction, leading to recombination of the excited charge carriers [48]. However, drastic and rapid progress in single-layer-device efficiency has been achieved (0.7%). In this case, the Schottky-barrier effect was enhanced by constructing organic layers sandwiched between a metal–metal oxide (Metal-Insulator-Semiconductor-MIS-device) [32].

### 3.2. Bilayer Device

**Figure 8:**
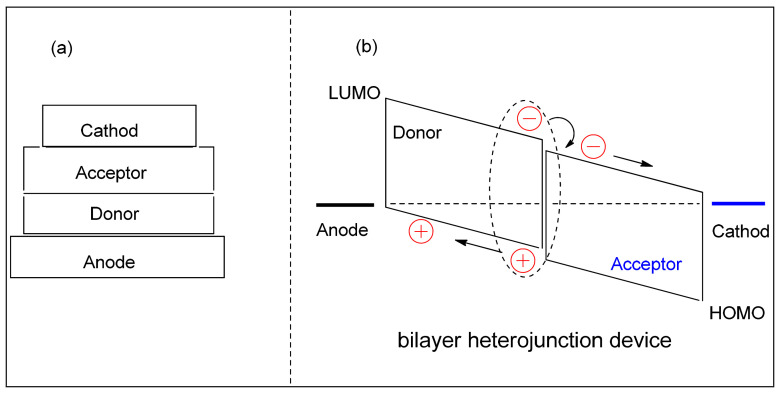
(**a**) Schematic of a bilayer organic structure. (**b**) Schematic of the phase separation of a bilayer device. The donor contacts the higher-work-function electrode and the acceptor contacts the lower-function electrode.

The introduction of the bilayer concept was considered a major breakthrough in the performance of electronic devices [32,46]. This type of device was introduced by Tang et al. in 1986, and this structural configuration proved to be the outstanding benchmark in the field of organic solar cells [49] to overcome the above-mentioned serious limitations of a single device [50]. Tang reported a two-layer device containing phthalocyanine derivatives as the p-type and perylene derivatives as the n-type components sandwiched between two electrodes as schematically depicted in Figure 8. The p–n junction device achieved a power conversion efficiency (PCE) of about 1% [49]. In this device, the p- and n-types are layered together with a planar interface as phase separation, which is key [49,51,52] in controlling the photovoltaic properties of the device [46]. Tang described that excitons generate and diffuse at the D–A interface where excitons are separated to free charge carriers (free hole and electron). The p-type (Donor) carried the holes to the prospective anode (indium tin oxide ITO) while the n-type (Acceptor) carried the electrons to the respective cathode (Al) [49]. A big advantage of this mode of operation and geometry was efficient exciton dissociation across the interface, resulting in a decrease in recombination losses [48,49,53]. However, a short distance of exciton diffusion into the bilayer was observed due to the excitons’ limited lifetimes. This limitation causes decay of the exciton to the ground state without reaching the acceptor domain. Moreover, the exciton diffusion length restricts the thickness of the active layer to a range of 10–100 nm, resulting in a loss of absorption of photons limiting the performance of the bilayer device [29,48,54]. Therefore, the interfacial area between donor and acceptor material should be as large an area as possible to guarantee better exciton dissociation, hence the invention of the BHJ concept [46].

### 3.3. Bulk Heterojunction (BHJ) Device

**Figure 9:**
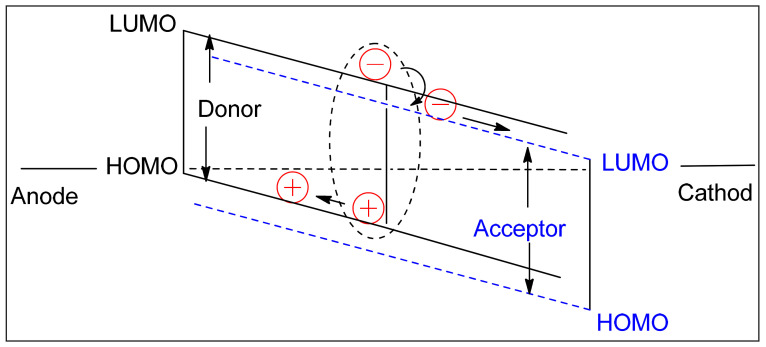
Schematic of a bulk heterojunction device. The donor component is blended with the acceptor component throughout the whole film. Consequently, photogenerated excitons can be separated into charge carriers at any place within a thin film.

Yu et al. [29] introduced the concept of the BHJ device; it was constructed by blending two organic materials, i.e., a conjugated-polymer donor and fullerene-derivative acceptor. Under this concept, the most common method of the formation of the active layer is to disperse the acceptor component in the conjugated polymer. The method is achieved by spin-coating a solution of both components in an organic solvent to form an interpenetrating network known as a BHJ [32,55,56]. This approach highly distributes and maximizes the donor–acceptor interfacial area within the active layer ensuring efficient exciton dissociation over the whole extent of the active layer, thus maximizing the number of free charge carriers. Moreover, this architecture secures the charge-percolated pathways to collect free charge carriers in the donor and acceptor at respective electrodes, completing the conversion of photon energy into electrical energy [28]. A typical dimension of the hetero-junction interface must be within the exciton diffusion length (10–20 nm) in order to obtain better exciton dissociation and high free-charge concentration. An efficient photo-induced electron transfer in conjugated polymer: fullerene composites can be accomplished by controlling the phase separation between the components using intimate mixing of morphology. This leads to enhancement of the efficiency of exciton splitting and transport and will be discussed further below [29,34]. Concerning BHJ device energy levels, the excited electron shifts from the polymer LUMO to the fullerene LUMO reaching the cathode via electron paths, while the hole transfers directly from the polymer HOMO to the prospective anode via hole paths. The potential differences between the two components at the interfacial area generate the driving force facilitating exciton splitting (bonded electron–hole pair) [57,58], as depicted in Figure 9.

**Figure 10:**
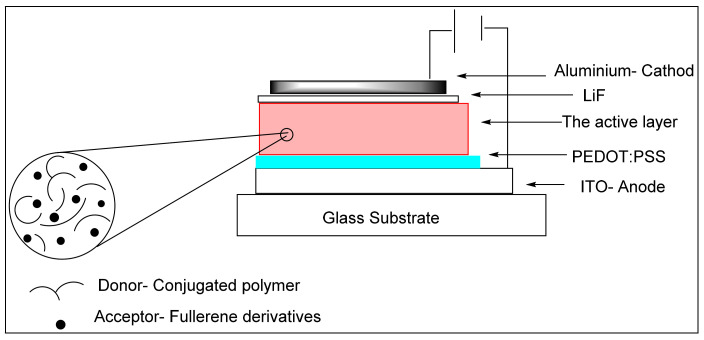
Schematic device structure of the OPV (glass/ITO/PEDOT: PSS/ active layer (polymer-fullerene bulk)/Li/Al).

The architecture of the BHJ has significantly improved the performance of the organic solar cells. However, there are substantial challenges to obtaining high efficiency, mainly because of the difficulty to dissociate the excitons (bonded electron–hole pairs) with the increased disorder of the morphology. Furthermore, inefficient physical mixing might create isolated regions within the BHJ layer [59]. The random distribution of electron-donating and -accepting materials within the single active layer leads to poor charge mobility due to complicated charge-carrier paths to the prospective electrodes [60], resulting in decay and recombination of charge carriers [48,60]. The layered structure in standard a BHJ device (Figure 10) consists of four essential layers. A conductive layer (polymer: fullerene mixture) is sandwiched between two electrodes, and a transparent ITO coated on a top of glass substrate is used as the anode. It is usually covered with poly(3,4-ethylenedioxythiophene) polystyrene sulfonate (PEDOT: PSS) which is applied between the photoactive layer and the anode, then a metal cathode such as Al is deposited as the top electrode on the OPV device. Both of the electrodes are connected into the internal circuit to flow photocurrent [28,46].

**Figure 11:**
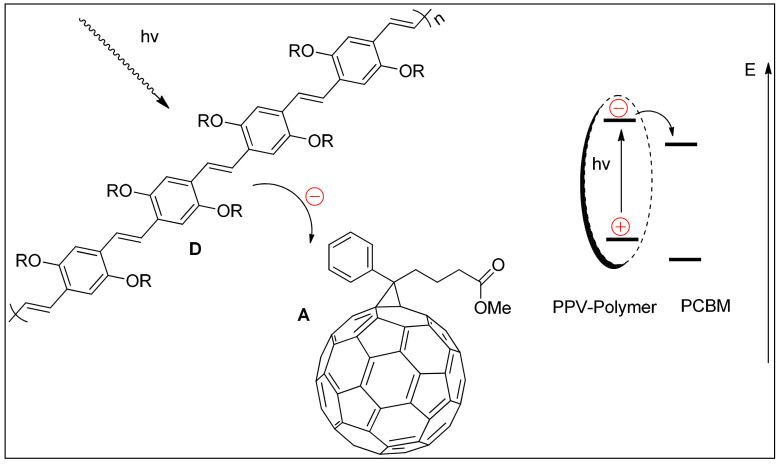
Illustration of the main process of photoinduced electron transfer from the donor (RO-PPV) to the acceptor (PCBM) as well as energy-level diagram of the working process for a D–A heterojunction solar cell.

Employing the conjugated polymer: fullerene derivatives in a bilayer structure achieved highly increased photoconductivity. Recently, Seok et al. reported highly efficient devices using a carbazole-based polymer/fullerene PC_70_BM. The device achieved a PCE of 7.12% [61]. Regarding the BHJ device components, buckminsterfullerene C_60_ and its derivatives such as [6,6]-phenyl-C₆₁-butyric acid methyl ester PC_61_BM have been intensively used as a standard acceptor in BHJ solar cells along the conjugated polymer as typical donor [29]. The exciton dissociation rate of the photo-induced electron transfer takes place rapidly, facilitating the charge splitting at the donor–acceptor interfacial area as shown in Figure 11. Other fullerene derivatives such as C_70_ and its counterpart C_71_ are the new standard n-type materials within active layers due to their solubility in organic solvents and absorption ability in the visible area [28]. There is still a need to further enhance the p-type material toward an ideal donor; various conjugated polymers have been employed to fabricate the active layer for electronic applications. Using different kinds of conjugated polymers allows the design of polymers with promising properties in devices [29]. Sprau et al. developed co-polymers with alternating benzodithiophene–thienothiophene and achieved a BHJ solar cell with a high performance of 9.5%, using the fullerene (PC_71_BM) as acceptor into the active layer [62].

Recently, the PCE of organic photovoltaic devices has enhanced rapidly with the milestone value that reached to 18%, due to the development of novel acceptors (non-fullerene acceptors, NFAs) instead of PCBM in traditional organic solar cells as well as matching polymer donors. The molecular structure of a polymer as donor domain mainly determines its morphological and optoelectronic characteristics of the OPV, which affects the photovoltaic performance and the ultimate PCE of the OPV device [63]. Many studies have investigated the effect of non-fullerene acceptors on OPV device performance. Young and his group developed an organic bilayer device through design and fabrication of flexible OSC models using SMD2 as donor and NFAs as acceptor. Despite having a deep (HOMO) level, the mixture of SMD2/NFAs exhibited remarkably promising performance in photovoltaic devices with a high PCE of 11.3% [64]. The organic solar cell (OSC) containing a new (PBDB-T-SF) polymer donor and a new small molecule (IT-4F) of non-fullerene as acceptor was introduced by Zhao et al. The study revealed that a PBDB-T-SF:IT-4F-based OSC device achieved a high efficiency of 13.1%, and an efficiency of over 12% which can be obtained with a thickness of 100–200 nm, suggesting the promise of NFA-based OSCs in practical applications [65]. Another research group synthesized and designed two chlorinated-thiophene-based polymers using NFAs, 1,3-bis(5-bromothiophen-2-yl)-5,7-bis(2-ethylhexyl)benzo [1,2-c:4,5-c′]dithiophene-4,8-dione (BDD) unit into each backbone, namely, P(F–Cl)(BDD = 0.2) and P(Cl–Cl)(BDD = 0.2), which showed high PCEs of 12.7% and 13.9%, respectively [66]. Another study revealed that the fine modification of the flexible side chains of NFAs as acceptor such as BTP-4Cl-12 conducted on a thiophene-based polymer such as PBDB-TF as donor achieved a high device performance efficiency of PCE = 17%. The optimal non-fullerene acceptor has a sufficient solubility and thus a favorable morphology, resulting in optimization of the chemical structures of the OPV that can improve device performance [67]. Lately, high performance of OPV device has been achieved due to fine-tuning and adjustment of the electronic structures and morphologies of the single junction of active layers. Yong et al. reported highly efficient devices using a new wide-band gap polymer (PBQx-TF) as donor and a new low-band gap of NFAs as acceptor. Incorporation of a third component such as NFA F-BTA3 in binary cells was an effective strategy that exhibited a high PCE of 18.7% [68].

## 4. Basic Operating Principles of Organic Solar Cells

**Figure 12:**
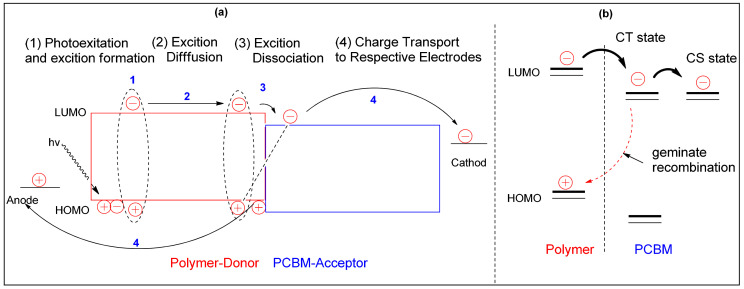
(**a**) Schematic diagram demonstrating the operating mechanism of a donor–acceptor BHJ device. (**b**) Basic energy level diagram for the photovoltaic process.

Commonly, the organic photovoltaic device is not able to generate free carrier charges (holes and electrons), unlike the inorganic semiconductors such as silicon solar cells [69]. The photo-excitation process in organic photovoltaic devices (Figure 12a) generates excitons (a coulomb-correlated electron–hole pair). Therefore, the organic semiconductors need an additional thermodynamic driving force to overcome the Coulombic attraction and split the excitons into free charges at the interfacial area [70,71]. It is well known that the organic photo-conversion system consists of two different potential semiconducting materials that are blended together to secure the driving forces for exciton splitting as a result of different electron affinities [72].

The operating principle of an organic photovoltaic device for completing the conversion of a photon of light into electrical energy is outlined in Figure 12b. The main mechanism of operation of an organic device comprises several steps including photo-excitation of the conjugated polymer (primary absorber) to generate excitons (electrostatically coupled electron–hole pairs). An electron can be excited from a low energy level to a high energy level in the donor domain of the light-capturing film in order to form the exciton due to photo-absorption. The photo-generated exciton then moves to a region where exciton dissociation takes place at an interfacial area as a result of a chemical potential gradient [73]. Previous literature has shown the photo-generated excitons possess a short lifetime and a diffusion length of 10–20 nm [74]. The formation of the charge transfer (CT) complex is preferred to take place at a hetero-interface when the binding energy of the complex is less than the energy differences between the LUMO energy levels of the conjugated polymer–fullerene [73]. However, the overall mobility of electron–hole pairs are limited to a few nm owing to the very small lifetime of excitons [75].

The CT state energy relies on the Coulombic attraction of the exciton that formed across at the donor–acceptor interface when the electron is transferred to the acceptor. The CT complex can convert to a charge-separated state (CS) or free charge carriers via exciton dissociation [73,76,77]. Generally, the exciton dissociation is highly dependent on the presence of a potential difference between p-type and n-type domains. It is desirable for the electron donor to have a high ionization potential (IP), while the electron acceptor has a strong electron affinity (EA). This difference can generate the driving force for the dissociation of the photo-generated excitons [75,77,78]. Furthermore, exciton dissociation can be enhanced by maximising the polymer–fullerene interfacial area, resulting in maximising the number of free charges. Optimising the morphology of the device also enhances the exciton splitting [28]. However, the recombination of geminate pairs across a hetero-interface area is assumed to occur as an undesirable outcome in the organic photovoltaic device. This can be attributed to failure of the exciton to reach the interfacial area within its lifetime; therefore, it will be considered as a loss mechanism and efficiency as radiative decay [75,77,78]. Once the photo-generated excitons have reached the interfacial area, they are dissociated to generate the free charge carriers as a sequence of electron transfer from the LUMO of the polymer (p-type) to the LUMO of the fullerene (n-type), whilst the holes are localized in the polymer component [70]. The transportation of free charge carriers through p- and n-type components to prospective electrodes for collection is essential to allow current extraction; both electrodes are connected with to external circuit to transport the free charges after migration through the materials of the active layer [75,77,78]. It is worth mentioning that intramolecular charge transfer (ICT) over the backbone of the polymer induces the charge to migrate through the polymer area [79]. In a BHJ configuration, interpenetrated pathways are important for charge transport. The efficiency of charge transport can be considerably affected by the molecular orientation, packing structure, and degree of structure ordered [73]. An internal structure and material design are the key features that determine photovoltaic performance of PSC through minimization of the energy required and charge loss mechanisms [73,78].

## 5. Characterization of Organic Solar Cells

**Figure 13:**
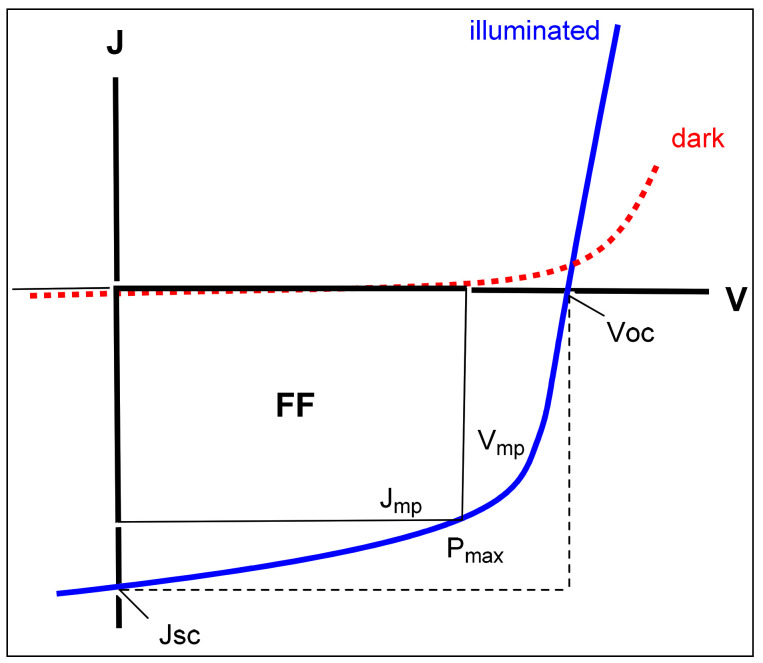
Common current-density–voltage (*J*–*V*) curve under dark and illumination for a typical organic solar cell.

Solar-cell performance can be evaluated by current (*J*)–voltage (*V*) curves. Typical of *I*–*V* characteristics for solar cells in the dark and illumination are depicted in Figure 13. Commonly, PCE represents the integrated evaluation of the performance of the electronic device [80]; this measurement is strongly dependent on key parameters including the short circuit current density (*J_sc_*), open circuit voltage (*V_oc_*), and fill factor (FF) [77,80]. The *J*–*V* characteristics are carried out under dark and illumination conditions with standard test conditions (STC) as laboratory testing. The STC is accompanied by solar spectrum intensity at 100 W/cm^2^ as well as an air mass (AM 1.5) at an incident spectral angle of about 48° and constant room temperature 25 °C [34,46,78]. Importantly, the standardized measurement condition used for organic solar cells is essential to be able to compare the parameters measured for the performance of devices and unify measurements across the world [32,34,46,81]. The graph can provide intrinsic information on how the OPV works and if the energy levels match with the organic material of the active layer, all these parameters will be helpful in the design of new materials [81].

The following critical parameters are used to determine the performance of organic solar cells:

Open circuit voltage (*V_oc_*): This parameter represents the maximum excluded voltage across the device when there is no current flow. It is highly dependent on the difference between the HOMO of the p-type material and the LUMO of the n-type material as illustrated in Equation (1) [73,78,80].

(1) $$Voc=\left(1/e\right)\;(|\;E^{Donor}\;HOMO\;|-|E^{Acceptor}|\;)-0.3\;V$$

where *e* is the elementary charge. A minimum energy difference of roughly 0.3 eV between the LUMOs of the conjugated polymer and fullerene is required to facilitate exciton dissociation and formation of free charge carriers [80]. Theoretically, a polymer with a low HOMO level introduces a high value of *V_oc_*. Nevertheless, continuously dropping the HOMO level of the polymer would inevitably increase the EBG of the conjugated polymer, resulting in a low *J_sc_* value, which is thought todiminish the absorption ability of the donor. Furthermore, the morphology of the active layer of devices has a noticeable influence on the *V_oc_* value [28].

Short-circuit current density (*J_sc_*): It is defined as the maximum current flow from a photovoltaic device when there is no applied voltage between the electrodes [73,78,80]. The *J_sc_* value can be affected by different factors including the amount of generation and collection of photo-generated charge carriers. Moreover, it is highly sensitive to the morphology of the active layer in devices, which affects exciton diffusion, dissociation efficiency, and charge-splitting ability [34,73,78]. Therefore, optimisation of photovoltaic devices is required through the choice of casting solvent, additives, and deposition method [32,46,82]. The *J_sc_* is correlated with the amount of light absorbed; hence, the absorption of the active layer, especially that of the conjugated polymer, should overlap with the solar spectrum in the visible area, where most of the spectrum energy (almost 70%) is distributed. Ideally, the conjugated polymer should absorb over a wide spectrum and have a good harvesting ability which is achieved by designing low-EBG polymers [28].

Fill Factor (FF): It is defined according to Equation (2) as the ratio of maximum actual power output (*J_mp_*, *V_mp_*) of the cell to its theoretical power output depending on *J_sc_* and *V_oc_.* This parameter represents the squareness of the *J*–*V* curve. It provides a good indication of how easy or difficult the charge carriers amount to be excluded out of a photovoltaic device to the electrodes is [78,81,83]. The parameter is sensitively affected by the charge mobility, the active layer thickness, and morphology [80].

(2) $$FF=\frac{P_{max}}{J_{sc}V_{oc}}=\frac{J_{mp}V_{mp}}{J_{sc}V_{oc}}$$

Understanding of FF and the fourth-quadrant *J*–*V* curve are important to probe what is happening inside the cell and how the device works, especially regarding the lifetime efficiency and mobility of charge carriers [81].

Power conversion efficiency (PCE): It is described as the percentage associated with the ratio of maximum produced power output (*P_max_*) by organic cells to the power input (*P_in_*) available in the incident light. This quantity measures the efficiency of OPV under standardized conditions [46,73]. Once the parameters have been identified, the PCE can be calculated using the following:

(3) $$PCE=\frac{J_{mp}V_{mp}}{P_{in}}=\frac{FF\;J_{sc}V_{oc}}{P_{in}}\times 100$$

Over the past few years, the power conversion efficiency (PCE) for BHJ organic solar cells has been steadily improved [81]. Recent theoretical calculations show that it is possible to achieve a PCE for BHJ solar cells over 12%, if the optimum polymer is available within the active layer [28,29], as a result of optimization of the nanoscale morphology of the active layer in photovoltaic devices [84]. It is required to obtain and combine all the impressive values such as *J_sc_*, *V_oc_*, and FF in one conjugated polymer in order to achieve the highest possible performance [28]. Recently, interesting studies were achieved by Zang et al. where they synthesized and designed a novel conjugated polymer that displayed a high PCE of 9.0% [85]. Another study by Kim et al. achieved a high PCE of 9.21% using a PT-ttTPD/ fullerene as BHJ device [86].

## 6. Molecular Engineering of Conjugated Polymers

**Scheme 1:**
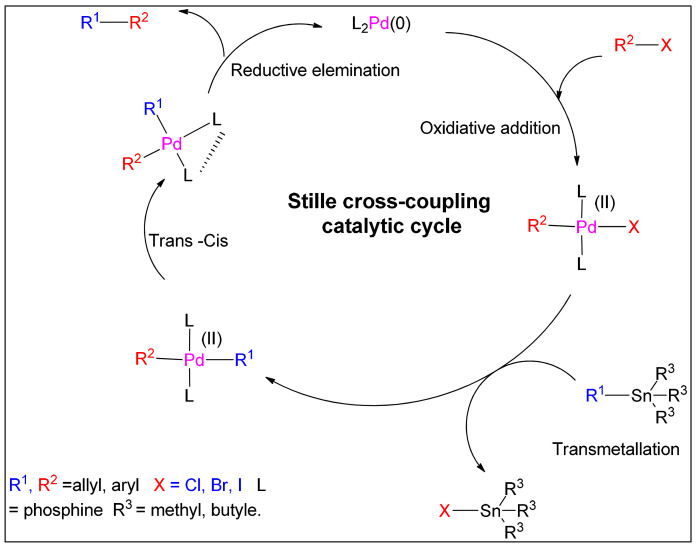
The suggested catalytic cycle mechanism for Stille coupling reaction.

Design and synthesis of conjugated polymers toward ideal p-type materials are the most critical challenges in order to obtain the most desirable characteristics. The harvest absorption qualities of the polymer in the visible area, high charge mobility, and its HOMO and LUMO positions can be considerably influenced by the make-up of the conjugated polymer. Understanding of molecular design by using various materials and different synthesis methods allows improvement of intrinsic photovoltaic properties of conjugated polymers and hence optimization of the overall PCE of organic devices. The chemical and optoelectronic properties of the conjugated polymer are dictated by the molecular structure. Thus, tailoring the molecular structure of the p-type material is fundamental. Furthermore, controlling morphology in BHJ devices is another parameter that contributes to film-forming properties, excitation–diffusion length, and charge transportation. All these factors are altered by the design of organic semiconductors. Both design and synthesis parameters are linked as depicted in Scheme 1 [29,87].

### 6.1. Requirement of EBG and Energy Levels

**Figure 14:**
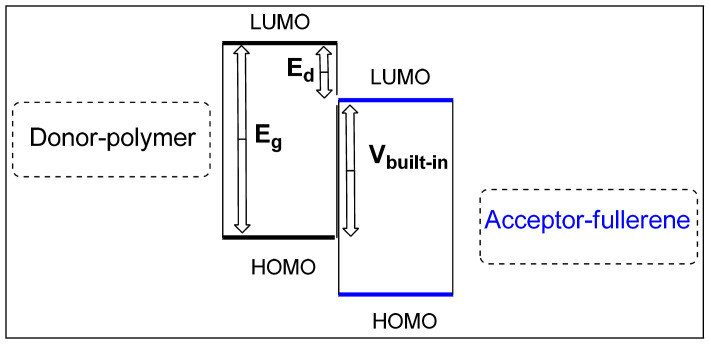
Schematic energy-band diagram of a simple BHJ solar cell consisting of polymer/fullerene derivatives.

The magnitude of the energy gap and energy level positions of HOMO and LUMO in conjugated systems of polymer dictate the optical and electronic properties, and hence influence the ultimate photovoltaic performance of PSC. To fully exploit sunlight energy, the conjugated polymer in the active layer should be compatible with the incident solar spectrum. Introduction of low-EBG polymers is required for efficient harvesting of the photo flux of sunlight [32,88]; thus, the amount of absorbed photons is determined by the size of the optical band gap of the polymer [89]. Therefore, development of narrow-EBG conjugated polymers for electronic applications is highly needed to absorb the greater part of the terrestrial solar spectrum in the visible region and maximize exciton generation [28]. Numerous chemical structures, with structural modifications and manipulations, have widely been studied in order to achieve low-EBG systems without the need for doping these materials [29] The main motivation of elimination of the doping process for the preparation of organic semiconductors is the poor solubility and infusibility of a given doped polymer for electronic applications [90]. A reduction in the EBG of conjugated polymers is obtained by either lifting the HOMO energy level, lowering its LUMO level, or compressing both of them simultaneously [29]. In the case of BHJ solar cells, the polymeric material (p-type) acts as the main light absorber in the D–A blend of the active layer [28]. Nevertheless, the properties of the n-type fullerene materials are still under consideration in order to increase their absorption ability and enhance their charge separation at the interfacial region upon replacing C_60_ with PCBM in the resulting active layer [1,29,91]. It has been mentioned above that *V_oc_* is theoretically linked to the difference in energy between the polymer HOMO level and the fullerene LUMO level in BHJ cells. This parameter is linearly based on the magnitude of the built-in potential as a result of the differences in energy levels in the polymer/fullerene active layer as shown in the energy diagram (Figure 14) [29].

In terms of energy, lowering the polymer HOMO level would inevitably achieve high *V_oc_* and enlarge the EBG of the polymer, leading to decreased *J_sc_* values because of diminishing of light-harvesting ability of the polymer. At the same time an increase in the polymer HOMO level would lead to decreased EBG of the polymer, resulting in a broad absorption spectrum and reduced *V_oc_*. Essentially, the difference between the polymer LUMO and the fullerene LUMO must be ~0.3 eV in order to facilitate exciton dissociation; thus, this guarantees the downhill driving force between both of the components of the active layer [28,92]. Lowering the polymer LUMO level for constructing a narrow EBG would significantly affect the efficiency of exciton splitting and charge transport, which eventually impedes the driving force at the D–A interface. Consequently, substantial efforts are being directed to balance the trade-off between EBG and energy levels of polymer/fullerene mixtures by manipulating the molecular structures of the polymer and using a variety of materials to obtain desirable performance in devices [92].

### 6.2. EBG Engineering of Conjugated Polymer

Intensive research has concentrated on the synthesis and design of conjugated polymers for fine-tuning energy levels and EBG in order to obtain desirable photovoltaic performance for integral success of PSC. Desired properties of conjugated polymers can be fulfilled by minimizing EBG and optimizing processability. The most important parameters that play a crucial role in the control of EBG polymers are: (i) Resonance effect, (ii) Bond length alternation (BLA), (iii) substitution/fusion effect, (iv) D–A alternation [29,90]. Apparently, the electronic applications of π-conjugated systems require a special combination of properties via a variety of materials. Regarding EBG engineering, it requires not only low potential EBG energy as the main target, but also more complex prerequisites that can be under consideration. Generally, most of the skeletons of conjugated polymers are derived from aromatic and heteroaromatic units such as PP, PPV, and PT. These structures show two types of resonance forms: aromatic and quinoidal. The quinoidal structure is energetically less stable than the aromatic form and hence has a lower energy and low EBG, resulting in its delocalization feature destructing aromaticity and thus contributing to decreased EBG, whilst the stability of aromatic structures tends to confine the π-electronic delocalization along the polymeric chain within aromatic rings due to resonance effects and hence reduce the delocalization. It is important to understand how synthetic strategies used affect the resulting low-EBG systems [29,93,94].

**Figure 15:**
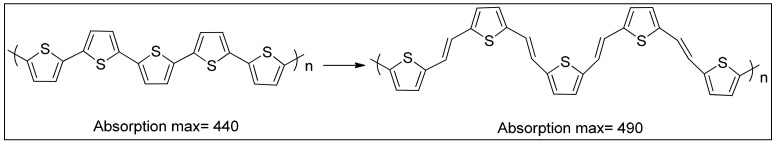
The chemical structure of PT polymers, showing the effect of adding ethylene bonds between the thiophene units in the molecular chain.

The BLA parameter presents the fundamental contribution to determining the magnitude of the energy gap of polymers such as polyene species. Additionally, inserting vinylene groups between adjacent units (Figure 15) over the chains of the polymer such as PT exhibits a useful strategy for reducin the EBG of the system due to dilution of the aromaticity [90,93]. This molecular manipulation used in the polymeric chains results in red-shifted absorption owing to an extension of the π-conjugated system [29,93].

**Figure 16:**
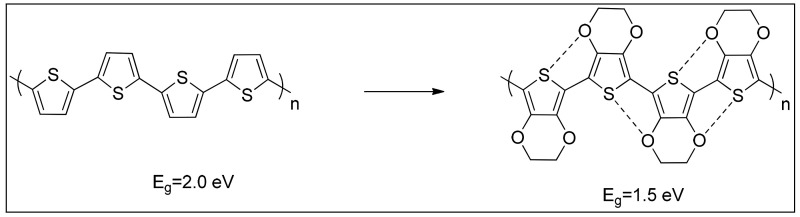
Intermolecular interactions between EDOT molecules.

The introduction of electron-deficient or electron-rich substituents is another way of tuning the HOMO and LUMO energy levels and EBG of a polymer. This approach (Figure 16) is beneficial in designing low-EBG polymers as well as in improving the internal interaction in the molecule such as 3,4-ethylenedioxythiophene (EDOT), resulting in enhanced planarity of the main structure owing to extension of the π-conjugated system and reduction in BLA [29,90,93].

**Figure 17:**
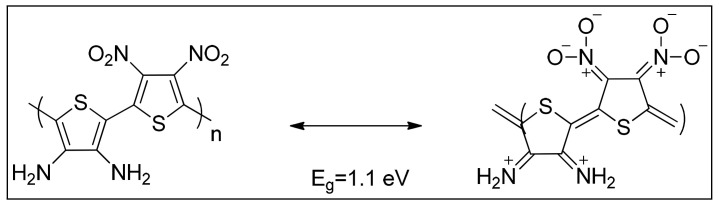
Incorporation of electron donor and acceptor on the thiophene molecules exhibits QD structures.

Furthermore_,_ a conjugated polymer (PT) containing electron-donor and -acceptor moieties as alternate repeat units (Figure 17) exhibits a significantly low EBG of 1.1 eV. This can be attributed to an increase in the quinoid (QD) character and rigidity of the resulting polymeric chain [29].

**Figure 18:**
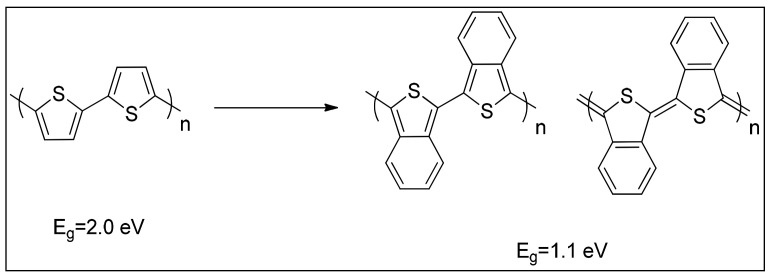
The molecular structure of incorporating a fused ring in the thiophene system.

The effect of extended aromatic π-conjugation using fused rings is an effective strategy to decrease the EBG of semiconducting polymers. This method would be helpful to increase the QD population over the main chain of polymers. In the case of PT, the fusion of benzene rings at the 3,4-positions of thiophene rings such as poly(benzo[c]thiophene) (Figure 18) introduces a low EBG of 1.1 eV instead of 2.0 eV for PT. The decrease in EBG arises as a consequence of direct de-aromatization of the thiophene ring and adoption of the QD structure [93].

### 6.3. Donor–Acceptor Alternation Approach in Conjugated Polymer

**Figure 19:**
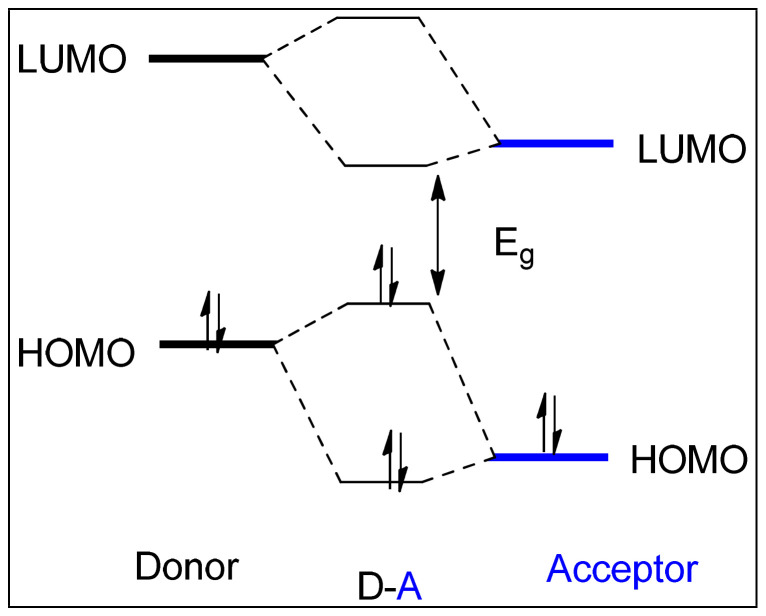
The energy diagram demonstrates the effect of polymerising electron-rich donor segments and electron-deficient acceptor moieties on the size of the EBG polymer.

This approach necessitates copolymerizing alternating strong electron-donor monomers with electron-acceptor monomers along the same π-conjugated system of the polymeric chain in a so-called D–A [80]. It became clear that alternating donor–acceptor segments contribute to a pull–push driving force between adjacent units in order to facilitate π-electron delocalization and the formation of a QD mesomeric structure (D-A 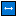 D^+^ = A^−^) over the polymer’s main backbone, resulting in reduced optical EBG [29]. Moreover, it is assumed that ICT between the donor-HOMO and acceptor-LUMO reduces the energy EBG of the polymer, leading to a significant reduction in the EBG owing to improved interchain delocalization. The decreased EBG energy can be rationalized and explained via Molecular Orbital Theory (MO). The HOMOs of the donor and acceptor segments (Figure 19) produce two new HOMOs due to the interaction of energy levels [29]. The interaction between low energy levels leads to broadening of the valence band. Similarly, the LUMOs of donor and acceptor moieties overlap to generate two new LUMOs of the D–A conjugated polymer, resulting in an increased magnitude of the conduction band after dispersing electrons to the new hybridized molecular orbitals. These energy levels can be modulated by various combinations of donor and acceptor segments into the conjugated polymer in order to tailor the polymeric characteristics for electronic application [89,90].

This synthetic route allows for obtaining well-engineered EBG conjugated polymers [90]. Numerous novel low-EBG alternating D–A conjugated polymers have been reported and afforded high performances of over 5% as polymer/PCBM from their BHJ solar cells. However, there are still challenges to obtaining desirable energy levels and decent EBG conjugated polymers with various structures used in repeating units [80].

### 6.4. Morphology of Active Layers in Organic Solar Cells

Optimizing the active-layer morphology is one of the most essential steps if high performance of the polymer is to be obtained. The nanoscale morphology ensures nanometer phase separation between donor and acceptor components in the active layer that enhance the exciton diffusion, dissociation, and charge transport [32,78,95]. Commonly, the ideal active layer morphology in BHJ devices should possess an interpenetrated network between the polymer and PCBM with a small domain size (10 nm), which is comparable to the exciton diffusion length and lifetime [78]. A previous study has shown that the short lifetime of the exciton requires short distances of diffusion (between 10 and 20 nm) [74]; this would facilitate all photo-generated excitons to reach interfacial area where they could dissociate into free charge carriers [32]. Maximizing the interfacial area between donor and acceptor within the active layer is a pre-requisite to optimize the dissociation of photo-generated excitons, so as to maintain bi-continuous phases in order to create charge pathways into the donor and acceptor domains to the contact electrodes [28,34,78]. It is clear that the active-layer morphology plays an important role in determining device performance and hence has a strong impact over device performance [46,77]. The microstructure and morphology of blends of polymer and fullerenes are directly affected by the processing steps, which include different parameters such as the casting solvent used, polymer/fullerene concentration and ratio, thermal and solvent annealing approaches, and additives. All these parameters manipulate and modify the active layer morphology in order to achieve better performance of the device. Spin coating is a widely used method in the laboratory as a solution phase deposition [78]. Solvent choice is the most critical factor for film-forming properties of the resulting morphology. A proper solvent for processing allows diffusion of the fullerene (acceptor) component into the polymer matrix and increases the roughness of the separation of phases in smaller fullerene domains [32,46]. Many studies investigated the effect of concentration and ratio contents on device performance, and researchers have found that a high fullerene load with a good solvent for the polymer/PCBM system is needed for an efficient charge carrier transport. An intimate inter-mixing of the active layer for BHJ solar cells is required for nanomorphology, allowing more diffusion and miscibility of fullerene clusters within the polymer matrix [32]. The optimal morphology of the active layer presents a balance between promoting exciton dissociation at the D–A interface and transport of free charge carriers to the electrodes [78].

## 7. Synthesis of Alternating D–A Copolymers

It is important to focus on synthetic approaches that are used to produce conjugated polymers for photovoltaic applications in addition to highlighting the important building structure units that construct the necessary monomers [29]. Pd-mediated cross-coupling reactions for organo-compounds have been widely used as common synthetic routes for preparing some new, efficient conjugated D–A polymers. They are various metal-catalyzed routes, including Stille, Suzuki, Sonogashira, and Heck coupling. Most highly efficient conjugated copolymers used in BHJ devices are synthesized via Suzuki and Stille coupling reactions using Pd catalysts. Notably, other reactions such as Sonogashira and Heck are seldom employed to prepare polymers with high efficiencies for organic solar cell applications.

### 7.1. Stille Coupling Reaction

The Stille coupling reaction is an efficient method that offers both high selectivity and versatility. It is tolerant towards most high functionalized molecules to form C–C bonds as result of coupling between halides and organo-stannanes. It is known that this reaction belongs to a family of palladium-catalyzed cross-coupling reactions. This reaction takes place under neutral reaction conditions in the presence of a palladium catalyst. However, one of the main drawbacks associated in this reaction is the high toxicity of organo-stannane compounds [96,97]. The suggested mechanism of this reaction is presented in Scheme 1. According to the proposed catalytic cycle, the oxidative addition of the aryl halide is the rate-determining step of the catalytic cycle to produce a *cis*-palladium complex. This intermediate product is subjected to *cis*/*trans* isomerisation to produce more a stable *trans*-intermediate product, followed by a transmetallation step, which involves reaction between the organo-stannane reagent and the Pd-complex to yield a *cis*-complex containing a new aryl. The last step is reductive elimination of the latter compound, which yields the target product and regenerates the Pd-catalyst again [98].

### 7.2. Suzuki Coupling Reaction

**Scheme 2:**
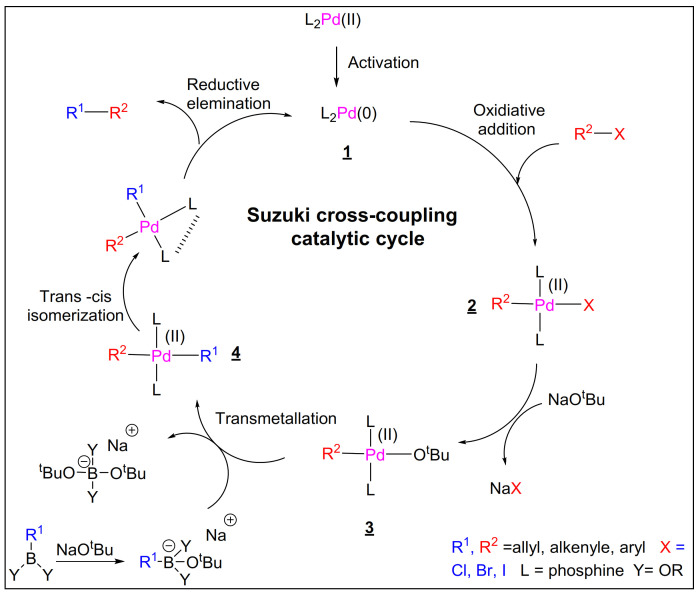
The proposed catalytic cycle for the Suzuki cross-coupling reaction.

The Suzuki coupling process was first described in 1979 by Akira Suzuki and his group [99]. It is the palladium-catalyzed cross-coupling reaction between organo-boronic acids or esters and halides in the presence of a base to form a C–C bond as outlined in the proposed mechanism (Scheme 2). In fact, the chemistry of Stille coupling is less complicated than the Suzuki reaction because the former reaction requires a base in order to create an intermediate product between the oxidative addition stage and the transmetallation step [97,100,101]. It is clear that the base plays a crucial role for the reaction to proceed forward and form intermediate compounds through the reaction, including the formation of a palladium complex, activation of the boronated reagent, and acceleration of the reductive elimination step [102]. However, boronic acids and their derivatives are less toxic compared to organo-stannane compounds. Furthermore, it is easy to remove unwanted by-products of boron compounds from the reaction mixture. Consequently, it is very common to perform this reaction on a large scale with mild conditions [97,101].

The first step is the oxidative addition of the palladium catalyst to the halide to generate an organo-palladium complex (**2**), followed by reaction with the base to produce an intermediate product (**3**). During the transmetallation step of the catalytic cycle, the ligands are transferred from the activated organo-borane reagent to the Pd(II)-complex formed to give the new Pd(II)-complex (**4**). Reductive elimination is the final step; in the reduction state the latter complex formed expels the desired product and the Pd(0) catalyst is regenerated [103].

### 7.3. Sonogashira Coupling Reaction

The Sonogashira coupling reaction is the palladium-catalyzed cross-coupling reaction between a terminal sp hybridized carbon of an alkyne and an sp^2^carbon of a halide to yield a variety of synthesized natural products and bioactive compounds [104]. The resulting products are employed in different applications such as the synthesis of oligomeric and polymeric materials. These materials are considered functionalized components for electronic and optical applications [105].

**Scheme 3:**
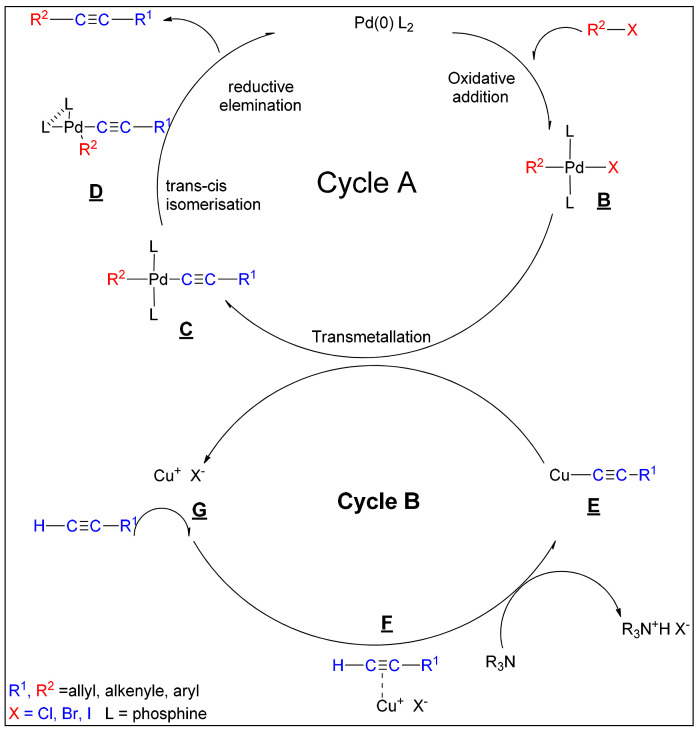
The general mechanism suggested for the Sonogashira cross-coupling reaction.

This reaction can be conducted easily under mild reaction conditions and treatment with a base that acts as solvent in the presence of Pd/CuI as catalysts. The co-catalyst in this coupling reaction increases the reactivity of the catalytic system. There is, however, one main complication associated with this type of cross-coupling reaction; the formation of homo-coupling alkyene is an undesired product through copper as co-catalyst in the presence of oxygen. To overcome this issue, it is essential to carry out the reaction under degassed and inert conditions to avoid acetylene dimer derivatives [104]. It is best to contemplate the catalytic cycle of the Sonogashira coupling reaction from the standpoint of the palladium catalyst as presented in Scheme 3.

The initial step is the oxidative addition of the halide to the Pd catalyst to produce a *trans*-intermediate (**B**); this step is the rate-limiting step of the catalytic cycle **A**. During this step, the oxidation state of the palladium catalyst changes from Pd(0) to Pd(II). The second step is the transmetallation step; in this step a copper acetylide species (**E**), which is generated via copper cycle **B** in the presence of the base, reacts with the Pd(II) complex formed to yield the ethynylene-based Pd(II) complex (**C**). During transmetallation, the acetylene groups are transferred from the copper acetylide species to give the Pd(II) complex (**C**). Prior to the reductive step, the *trans*-alkyne species is subject to *trans*-*cis* isomerization to yield *cis*-intermediate (**D**). The final step is reductive elimination to obtain the desirable alkyne and the Pd(0) catalyst is regenerated. Cycle **B** is less well-known and under debate. It is hypothesized that the presence of the base helps the formation of copper acetylide species involved in the transmetallation step of Cycle **A** with expelling the copper halide (**G**) to Cycle **B** again, followed by the addition of acetylene reagent to yield π-acetylene complex (**F**) [104].

## 8. The Use of Fused Rings as Electron Donor and Acceptor Units in Conjugated Polymers

The aim of using a few single aromatic units in new D–A polymers is to control the electron-donating ability of these units, which has influence on the HOMO energy levels and the EBG of the resulting conjugated polymers. Obviously, fused conjugated units such as fused three rings are employed to tune electronic properties and affect charge mobility. In addition, it is more likely to improve the intermolecular interactions of the main backbone of the polymers [28], resulting in improved hole mobility in polymers as a consequence of adopting more ordered structures [106]. The electron-accepting units are of equal importance in controlling the energy levels and band gaps of conjugated polymers to donor units. Notably, the acceptor unit plays a critical role in determining the electronic structure of the semiconducting material according to the strategy of alternating D–A polymers. Desirable narrow-EBG polymers require a deep LUMO energy level below −4 eV to ensure electron transport that is considerably influenced by the nature and structure of the acceptor unit [25,28].

### 8.1. Fluorene as Donor Unit in Conjugated Polymers

**Figure 20:**
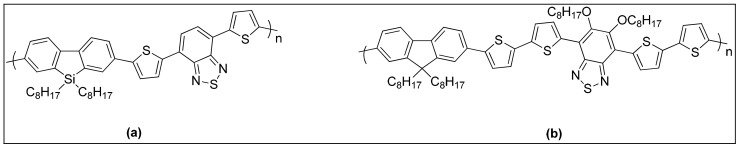
Molecular structures of: (**a**) P Si-FDTBT; (**b**) PFDT2BT.

The fluorine (FNE) unit is one of the most important polycyclic aromatic hydrocarbons and has intensively been studied as an electron-rich unit for electronic applications [107]. Its derivatives are commonly used to form either homopolymers (polyfluorene) or alternating copolymers. Utilization of FNE moieties in D–A systems enhanced the pull–push force in alternating polymers that demonstrated promising performance in organic photovoltaic devices. Furthermore, these species are useful due to unique features such as high thermal and chemical stability and accessibility of synthesis and alkylation as well as favorable charge (hole) carrier mobility. The chemical structure of FNE presents a central heterocyclic ring that would eliminate the further severe steric hindrance of benzene rings. In addition, the side alkyl chains can be attached at the 9-position of the FNE units to obtain high-molecular-weight polymers that can be produced without adding any steric hindrance on polymer chains [28,107]. Wanget al. synthesized pull–push alternating polymers of 2,7-silafluorene –SiF-and 4,7-di-2-thienyl-2,1,3-benzothiadiazole (Figure 20a) that showed a high power-conversion efficiency of 5.4% when fabricated with PC_61_BM in BHJ organic photovoltaic devices. Despite possessing a moderate optical EBG (1.82 eV) in the solid state, this polymer displayed good charge mobility in photovoltaic devices [108]. Another study developed a new pull–push polymer based on 9,9-dioctylfluorene (Figure 20b); this polymer showed excellent charge mobility and a maximum PCE of 6.2% when the polymer was fabricated with PC_70_BM in photovoltaic devices [109].

**Figure 21:**
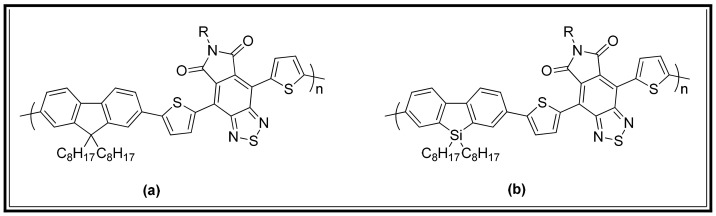
Molecular structures of: (**a**) PFDTBTDI-DMO and PFDTBTDI-8; (**b**) PDBSDTBTDI-DMO and PDBSDTBTDI-8.

Previous work in Iraqi’s group has synthesized four FNE and dibenzosilole-based copolymers by copolymerizing 2,7-fluorene and 2,7-dibenzosilole (DBS) with a new acceptor moiety, 4,7-di-2-thienyl-2,1,3-benzothiadiazole-5,6-*N*-alkyl-dicarboxylic imide (DTBTDI) (Figure 21) [110]. All polymers were synthesized in good yields via Suzuki polymerization. Dibenzosilole-based polymers have slightly lower optical band gaps relative to their FNE-based analogues. All polymers displayed deep-lying HOMO levels of 5.59 eV. 

### 8.2. Carbazole as Donor Unit in Conjugated Polymers

**Figure 22:**
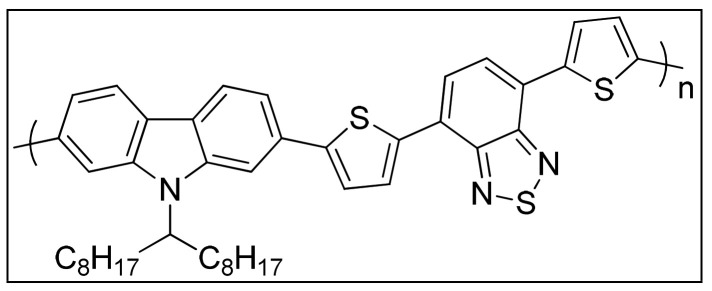
Molecular structure of PCDTBT.

The carbazole (CZE) molecule is the structural counterpart to FNE [111]. The presence of the nitrogen atom in its central fused pyrrole ring improves the donating ability of the unit skeleton, which provides CZE with desirable properties as a result of increased oxidative stability. In addition, functionalizing the nitrogen atom in the 9-position can bestow desirable solubility and physical properties of the synthesized polymer. Furthermore, the presence of the pyrrole fused ring results in a fully aromatic and electron-rich unit [112]. Poly(2,7-carbazole) derivatives have been studied intensively; these polymers displayed high hole mobility and deep HOMO levels, resulting in promising photovoltaic performance. It is well known that an ideal polymer for solar application devices should possess narrow EBG and tuning energy levels in order to broaden visible absorption. One of the most effective approaches towards an increase in the PCE in BHJ solar cells is to design alternating D–A copolymers in which the tuning of the electronic structure and low-EBG polymers can be obtained. The alternating copolymers based on 2,7-carbazoles have been reported as the donor moieties in D–A polymers for photovoltaic devices. It is believed that incorporation of the unique features of the CZE moiety will present a promising p-type material in PSC for high photovoltaic performance [113]. Recently, Chu et al. synthesized the D–A polymer poly[*N*-heptadecanyl-2,7-carbazole-*alt*-5,5-(4,7-di-2-thienyl-2,1,3-benzothiadiazole)] (PCDTBT) (Figure 22). This polymer demonstrated excellent charge mobility and oxidative stability, as well as a high efficiency above 7%, which was achieved when the polymer was mixed with fullerene derivatives in BHJ solar cells [114].

**Figure 23:**
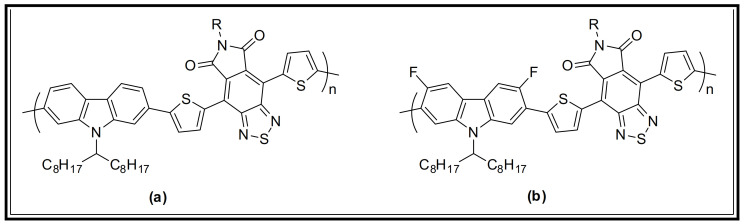
Molecular structures of: (**a**) PCDTBTDI-DMO and PCDTBTDI-8; (**b**) P2F-CDTBTDI-DMO and P2F-CDTBTDI-8 [109].

Iraqi et al. designed and synthesized four novel alternating copolymers, PCDTBTDI-DMO, PCDTBTDI-8, P2F-CDTBTDI-DMO, and P2F-CDTBTDI-8, via Suzuki polymerization. The polymers were prepared by copolymerizing 2,7-linked CZE units and 3,6-difluoro-2,7-linked CZE moieties flanked by thienyl units as electron-donor units and benzothiadiazole dicarboxylic imide (BTDI) as electron-acceptor units. All polymers were prepared in good yields and possess excellent solubility in common organic solvents (Figure 23) [115].

**Figure 24:**
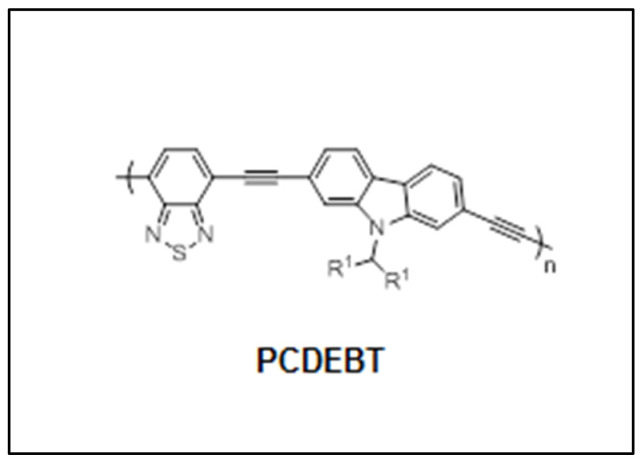
Molecular structure of PCDEBT.

Azzawi et al. prepared PCDEBT (Figure 24) via the Sonogashira coupling reaction comprising 4,7-linked benzothiadiazole (BTDZ) units and 2,7-linked CZE using ethynelyene (ETN) spacers between the donor and acceptor moieties over polymeric chains [116]. This study exhibited that incorporation of acetylene linkers between the BTDZ electron-accepting units and CZE electron-donor units over polymer chains lead to wide a EBG (optical EBG 2.2 eV, electrochemical EBG 2.35 eV), as a result of the electron-accepting properties of the acetylene units. This is a consequence of a decrease in electron delocalization and conjugation length between donor and acceptor moieties caused by the ETN linker. Furthermore, PCDEBT showed low yield and solubility in common organic solvents as a result of the introduction of ETN linkers over the main chains of the polymers, which will adopt more planar conformations.

### 8.3. Anthracene-Based Conjugated Polymers for Application in Solar Cells

**Figure 25:**
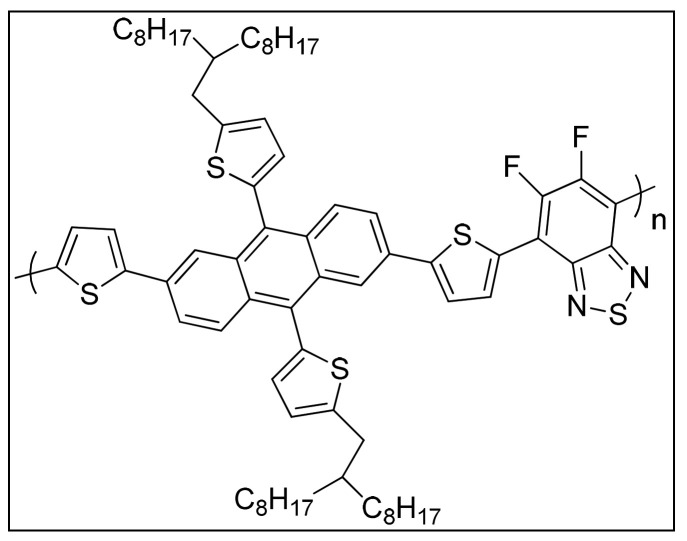
Molecular structure of PTADTDFBT.

The phenomenon of electroluminescence of anthracene (ATN) crystals was observed in the 1960s, and since then ATN and its derivatives have been widely studied and investigated in different applications such as OLEDs and OFETs. Great progress was achieved in the application of ATN systems due to their promising characteristics, such as good electrochemical properties and excellent charge carrier transport [35,117]. The electronic and photovoltaic properties of D–A polymers based on ATN are highly dependent on the nature of the ATN units and the positions through which these units are linked with electron acceptor units in D–A polymers. 9,10- and 2,6-linked ATN units allow quinoid character and extended conjugation systems. Some studies revealed that 9,10-linked ATNs in D–A polymers show low performance as a result of the twisting angle between the ATN rings and the adjacent units, which reduces the length of electronic conjugation over the main polymer backbone. Therefore, it is speculated that incorporation of 2,6-linked ATN-based conjugated polymers would lead to good electrochemical and photovoltaic properties [118]. It is worth mentioning that proper alkyl chains attached to ATN moieties present strong impacts on the π–π stacking over the polymer chains, resulting in excellent charge motilities and high performance of PV devices [119]. Egbe et al. incorporated acetylene spacers in a series of 9,10-linked ATN polymers. This study revealed that efficiencies ranging from 0.34 to 3.14% were obtained for the resulting polymers [120]. More recently, Jung et al. synthesized the pull–push with 2,6-linked ATN (PTADTDFBT) (Figure 25). Despite having a medium EBG, the resulting polymers showed a high *V_oc_* value of 0.97 V and *J_sc_* higher than 12 mA/cm^2^ in BHJ devices. PCE in excess of 8% has been achieved in these devices. This can be explained by the aggregation behavior of polymer chains in blends with PCBM for the ATN-based polymers as a sequence of strong π–π stacking, leading to a crystalline form of this polymer [121].

**Figure 26:**
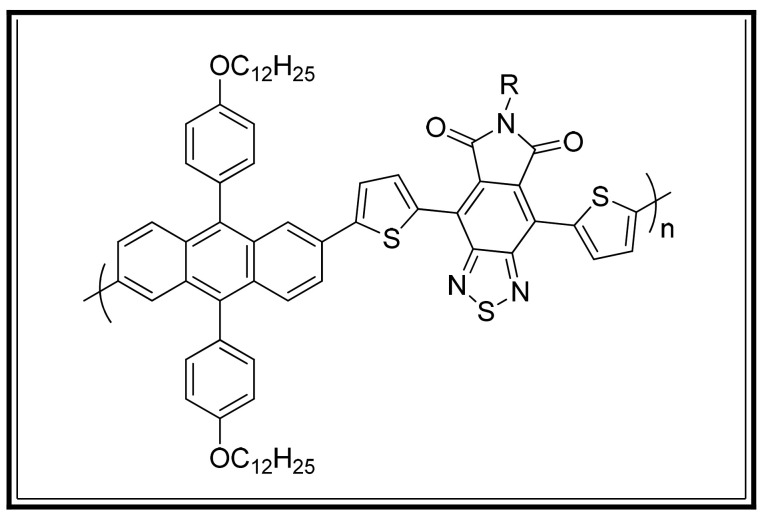
Molecular structures of PPADTBTDI-DMO and PPADTBTDI-8.

Two novel low-EBG D–A copolymers, poly[9,10-bis(4-(dodecyloxy)phenyl)-2,6-anthracene-alt-5,5-(4′,7′-bis(2-thienyl)-2′,1′,3′-benzothiadiazole-*N*-5,6-(3,7-dimethyloctyl) dicarboxylic imide)] (PPADTBTDI-DMO) and poly[9,10-bis(4-(dodecyloxy)phenyl)-2,6-anthracene-alt-5,5-(4′,7′-bis(2-thienyl)-2′,1′,3′-benzothiadiazole-5,6-*N*-octyl-dicarboxylic imide)] (PPADTBTDI-8), were synthesized by Iraqi and co-workers (Figure 26). The bis-boronate ester of 9,10-phenyl substituted ATN flanked by thienyl groups as electron–donor units copolymerized with BTDZ dicarboxylic imide (BTDI) as electron–acceptor units. Both polymers have comparable molecular weights and have a low *E_g_* of 1.66 eV. The polymers have low-lying HOMO levels of about −5.5 eV as well as similar LUMO energy levels of −3.56 eV [122].

### 8.4. Benzothiadiazole and Naphthothiadiazole Units in Conjugated Polymers

The BTDZ repeat unit is considered to be one of the classical strong electron-acceptor units used in D–A polymers for PV devices. The commercial availability and planarity of BTDZ units serves as a platform to use them in constructing low-EBG D–A copolymers for electronic applications [28,116,123]. Therefore, these units have attracted much attention in the research community for the development of narrow-EBG polymers in order to obtain promising physical and photovoltaic performance. However, the EBGs of BTDZ-based conjugated polymers, which range from 1.7 to 1.9 eV, are too high to harvest a large spectral portion of sunlight for PV applications as a result of the relatively weak electron-accepting ability of the BTDZ repeat unit compared to other acceptor units. Moreover, the rigid geometry of BTDZ units without alkyl chains results in limited solubility and low molecular weight of the resulting polymers, resulting in a negative impact on the fabrication and performance of the materials in organic electronic devices. Thus, the modification of its molecular structure to obtain stronger electron-accepting properties is an attractive way towards more efficient polymers for use in bulk heterojunction solar cells using fullerene derivatives as electron acceptors [123].

**Figure 27:**
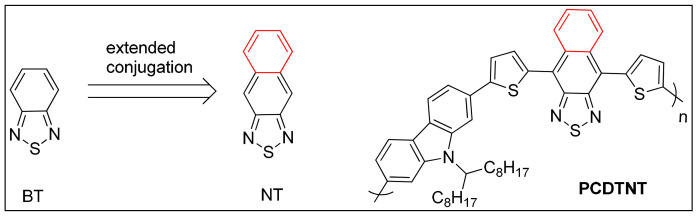
Molecular structure of BT and NT monomers as well as molecular structure of PCDTNT.

There are many approaches to preparing conjugated polymers with low optical EBG in order to expand the spectrum of their light harvesting of sunlight. One of the methods is to add a fused ring to the BTDZ unit. This modification has been reported by some studies via replacing BTDZ with naphthothiadiazole (NT) units in the alternating D–A backbone in conjugated polymers. The utilization of NT units into the polymer main chain would extend aromatic π-conjugation of NT. This acceptor unit possesses high electron-withdrawing capability, which provides deep LOMO levels and broad absorption. The incorporation of NT into the alternating conjugated polymer should lead to lower EBG and enhance interaction packing and thus promote strong π–π stacking conformations due to the more planar structure of NT units. The aim of employing strong-electron-affinity NT is to a build strong π–π stacking backbone in order to facilitate charge-carrier mobility over the structure. This can lead to high performance of conjugated polymers for PV devices [124]. Kim et al. developed a counterpart of BTDZ-based polymers by replacing BT with NT in poly(2,7-carbazole-*alt*-4,7-dithienyl-2,1,3-benzothiadiazole) PCDTBT (Figure 27). The new polymer exhibited good optical and electrochemical properties via low band gap (1.71 eV), which resulted in an increase in the HOMO energy level compared to its BTDZ polymer analogue. However, the polymer provided a low PCE of 1.31% in BHJ cells using PC_71_BM as an acceptor; this may be attributed to steric hindrance between NT and adjacent units that affected intermolecular interactions as well as an unfavorable morphology of the devices [125].

**Figure 28:**
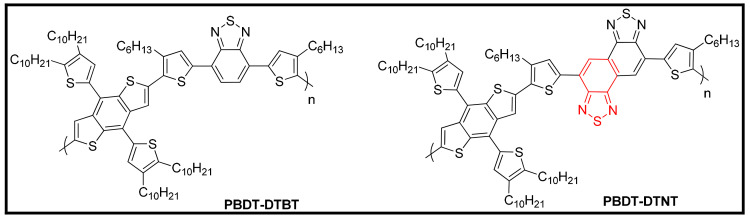
Molecular structure of PBDT-DTBT and its counterpart PBDT-DTNT.

Wang et al. investigated the effects of the incorporation of NT units instead of BT units on PBTT-DTBT to yield PBDT-DTNT (Figure 28). The NT-based polymer displayed a narrow EBG relative to that of the BTDZ-based polymer owing to changing of both HOMO and LUMO levels simultaneously. It can be seen clearly that the NT copolymer exhibited a significantly promising performance in photovoltaic devices with a high PCE of 6% when compared with its counterpart (PBD-DTBT) which provided a PCE of 2.1% [126].

**Figure 29:**
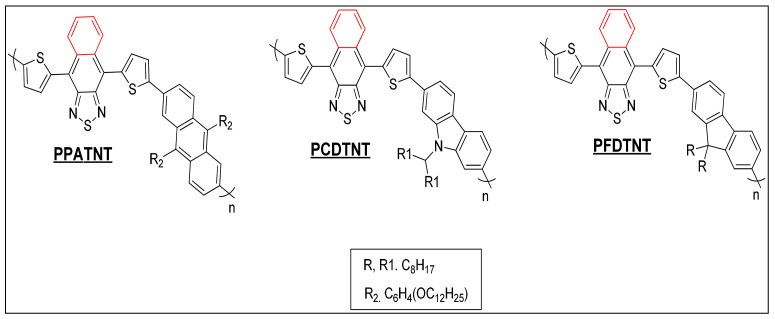
Molecular structure of NT-based polymers.

Azzawi et al. synthesized a new series of NT-based polymers (Figure 29) and compared them with their BTDZ counterparts in order to ascertain the effect of replacing 2,1,3-benzothiadiazole (BTDZ) with 2,1,3-naphthothiadiazole (NT) in this series of conjugated polymers. Incorporation of NT moieties instead of BTDZ moieties resulted in red-shifted absorption maxima and lower-EBG conjugated polymers as a result of a more extended electronic delocalization on the NT unit by virtue of an additional fused benzene ring in comparison with the BTDZ unit. Moreover, replacing BTDZ acceptor units with NT units over the main chain of polymers lead to polymers with increased molecular weights and solubilities, which was attributed to the twisting of polymer chains out of planarity as a result of steric hindrance between naphthothiadiazole units and adjacent thiophene rings [127].

## 9. Commercial Success of Organic Solar Cells

It is obvious that using alternative energy technologies such as solar cells will remarkably contribute to slowing climate change by reducing the level of CO_2_ emissions and resource utilization [128]. Solar power is considered one of various renewable energy sources that significantly contribute to solving environmental problems [129]. However, there are still limitations in using silicon-based solar cell technology in terms of energy efficiency and cost, as well as its long-term effects on the environment. Since organic photovoltaic (OPV) technology has potential advantages over traditional silicon solar cells, which include its lightweight nature, flexibility, good processability, solubility, and low manufacturing cost, so this new technology (OPV) has caught extensive attention of the solar cell research community, which has focused on developing this technology during the last few decades [130]. The printability of organic polymer means that it can be soluble and printed as ink from a printer much like those used to mass produce books, magazines, and daily newspapers. The printed solar technology displays promising photovoltaic performance that makes it a good candidate for a broad number of applications such as water pumps, disaster shelters, power for streetlights, camping equipment, and smart construction materials. It is worth mentioning that the printed technology is publically available to display at the CANOPY in Lane Cove on Sydney’s north shore. As well, the printed photovoltaic panels have been installed on covered walkways to generate power for a series of lights that come on at night. This novel approach was innovated by University of Newcastle Physicist Professor Paul Dastoor. Many life-cycle cost-analysis assessments have displayed that the printed OPV technology likely provides a cost-effective contribution to the world’s energy supply [128,129]. Furthermore, the PCE of OPV at a laboratory scale is now at 18%, which is equivalent to that of photovoltaic devices based on polycrystalline silicon structures [131].

Recently, the efficiency of polymer photovoltaic devices has been improved and increased through the development of novel approaches using different types of buffer layers of the OPV devices [132]. The main challenge for large-scale fabrication is that OPV technology must now fulfill a figure of merit (FOM) that combines both the synthetic complexity (SC) of the organic materials and the efficiency of the devices [133,134]. According to this viewpoint, the dangerous organic solvents utilized in OPV are restricted because of the environmental contamination risk and damage to human health. Consequently, this leads to greater SC and higher fabrication cost. As a result, scientific researchers who are attempting to industrialize OPV technology have been researching new environmentally friendly (“green”) solvents [135]. Pre-aggregation of the donor and acceptor polymer domains results in advantageous phase separation, which is similar to bulk heterojunction (BHJ) devices. Ideal morphological structures of OPV and environmentally friendly fabrication are both made possible by nanoparticle (NP) ink technology, which processes donor and acceptor materials to produce nanoparticles using green solvents such as water or alcohols [136,137].

**Figure 30:**
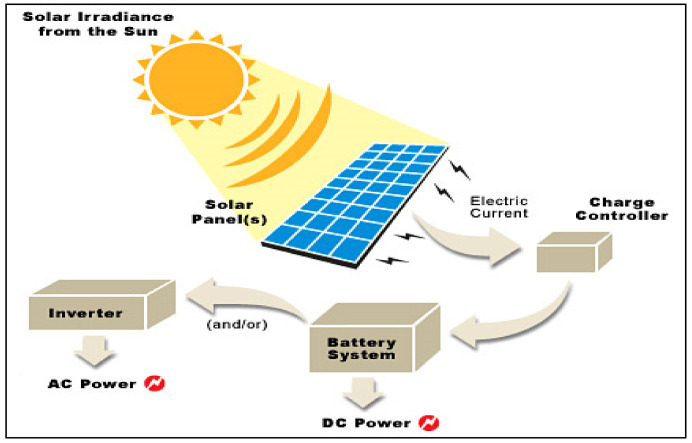
Schematic illustration for obtaining AC power from solar panels.

In the 21st century, one of the most crucial tasks is reducing carbon dioxide (CO_2_) emissions to zero in order to mitigate the consequences of global warming [138]. The integration of high-band-gap material with a front cell is considered an effective strategy to increase output voltage while maintaining current, which enhances the OPV ability, specifically designed interlayer, and a back cell with a low-band-gap material. This feature requires tuning of band gap and energy levels of polymeric materials that were explored for their chemical properties [139]. Yang and his group were able to fuse single cells into a “tandem” cell to harness the enhanced efficiency, occupying the same footprint but with a little more depth to make room for both of the “tandem” cells. These organic semiconductors, which can be synthesized at low price even in huge volumes, made it possible. Figure 30 presents a schematic representation of how AC power can be generated using solar panels. The packed cells employ various solar radiation wavelengths to make better use of harvesting sunlight. The “tandem” cell should function well and greatly enhance efficiency, which was previously not achievable due to the requirement of material and band gap matching. The efficiency of the “tandem cell” with the Japanese polymer increased from 8.62% to 10.6%, breaking the former record set by the “tandem technology” without it, which depended on various absorption light bands in the tandem cells. Sumitomo’s group created new polymeric materials that absorb the infrared (IR) wavelength and lets the cell benefit from that spectrum as well. Within the next few years, they intend to market this product using existing technology, with the ultimate objective of achieving 15% efficiency. This is a potential opportunity given the enormous sums of money spent on solar technology each year now [139].

**Figure 31:**
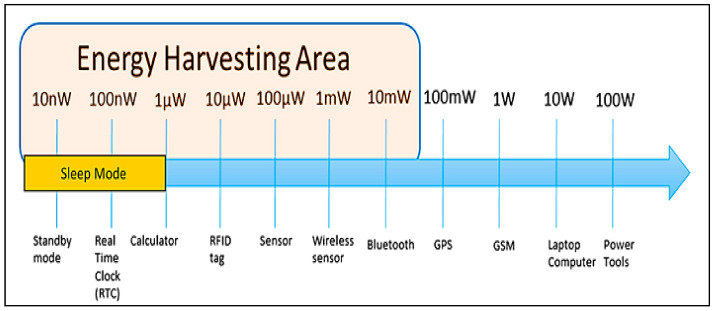
Levels of power for portable electronic devices and harvesting of energy capabilities of PV cells for indoor uses.

To achieve this within the time frame available for the 1.5 °C objective, optimization of efficiency and a rapid move away from fossil fuels in the transportation, electricity, and heating sectors in favor of sustainable technology will be fundamental [138,140]. Organic semiconductor photovoltaic devices have shown potential use for next-generation solar technology because of their distinctive features such as light weight, semitransparency, and affordable solution procedures [141]. Figure 31 shows the harvesting power of portable electronics and the energy-harvesting potential of PV cells for indoor applications.

**Figure 32:**
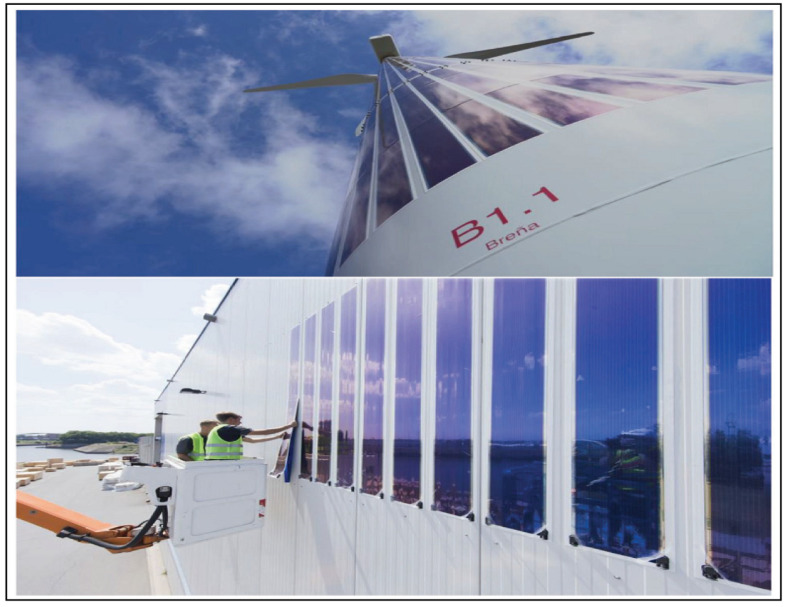
Heliatek’s solar panels on wind turbines. Bottom: ≈185 m^2^ of Heliatek’s solar panels applied on an industrial building in the seaport of Duisburg, Germany.

There are essentially two such niches: the first one is building-integrated photovoltaic systems (BIPV) [139,142]. The storage facilities including walls and roofs are covered with OSC in the most basic type of BIPV (Figure 32). Since these organic semiconducting structures are normally not designed to support heavier loads, OSC, a plastic foil weighing less than 1 kg/m^2^, is favored over conventional crystalline silicon devices, which typically weigh at least ten times more. Another advantage of OSC is its highly straightforward fixation: double-sided adhesive tape is utilized to simply install the OSC modules [139]. The short lifetime of OPV is less significant because small industrial buildings of this type are sometimes only built for 20 years of use. The integrated construction of semitransparent photovoltaic devices is a more cutting-edge application [143]. OSC is considered the best choice for this application since it gives semitransparency with lower losses in any cell than non-transparent cells and color-neutral absorption [144]. Traditional silicon solar cell technology can also be employed to create semi-transparent modules, for example by spacing out the cells. However, semitransparent OSC’s ability to make uniform semitransparency is superior [139]. BIPV markets only make up about 5% of the total PV market, which is a modest proportion. Although it is a very attractive investment that is expanding rapidly, it might yet reach a market size of over EUR30 billion in the coming years [145]. The second market is for consumer products that benefit from flexible properties, as OSC in clothes, bags, or camping equipment.

In addition to allowing the use of building-integrated photovoltaics, OPV technology is superior to traditional PV in that it can better meet the aesthetic and functional needs of architects and designers. OPV panels have been presented into several types of glass facades and membrane structures of architectures. One of these designs is the African Union Peace (AUP) and main Security Building in Addis Ababa, where OPV panels were installed to power lights throughout the structure, greatly lowering its thermal load. In 2016, OPVIUS GmbH and BGT Bischoff Glastechnik AG collaborated together under the supervision of Timo Carl Architecture in Kassel to create a more current example of OPV integration into building components. Another late example of OPV technology installation is the OPV facade created by SUNEW, Brazil. In the past few years, several electronic portable goods and wearables have been produced for commercial usage in parallel with these developments, creating the foundation for IOT technology.

**Figure 33:**
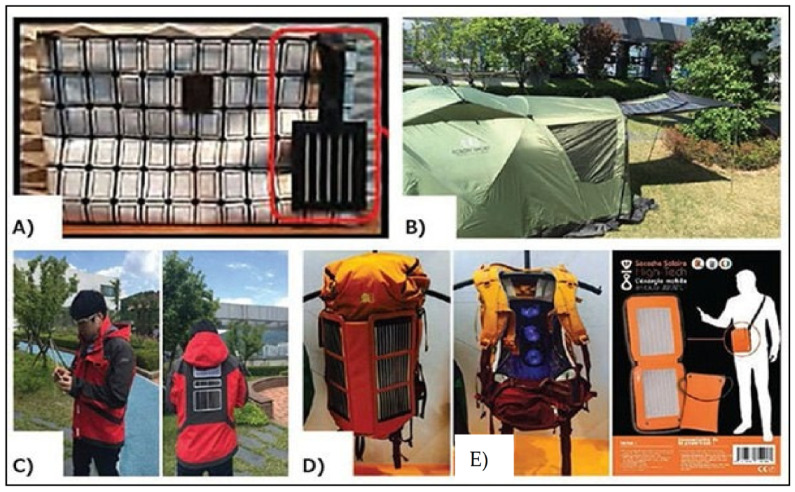
(**A**) Tailor-made smart bag by Kolon Industries, integrating an organic solar panel energy harvester (outlined with red rectangle) and NFC technology. (**B**) Star Tent by Kolon Industries, integrating LED lighting with OPV as the energy source. (**C**) Smart jacket integrating an OPV module, LED, and Bluetooth by Kolon Industries. (**D**) Backpack by Kolon Industries, integrating OPV as the power source for cooling fans. (**E**) Solar bag designed and manufactured by Armor using ASCA© photovoltaic films as the source of energy.

Markets where OPV technologies are expected to have an effect include healthcare services (wellness monitoring, diagnostics), augmented reality, cloths, fitness, and security. Commercial applications of OPV in outdoor gear, including tents, backpacks, and waterproof coats, are depicted in Figure 33B–D. Figure 33A depicts a smart bag powered by OPV that pairs with a smartphone through Bluetooth. The bag signals incoming calls and messages and sounds an alarm if the phone device is left behind [146].

**Figure 34:**
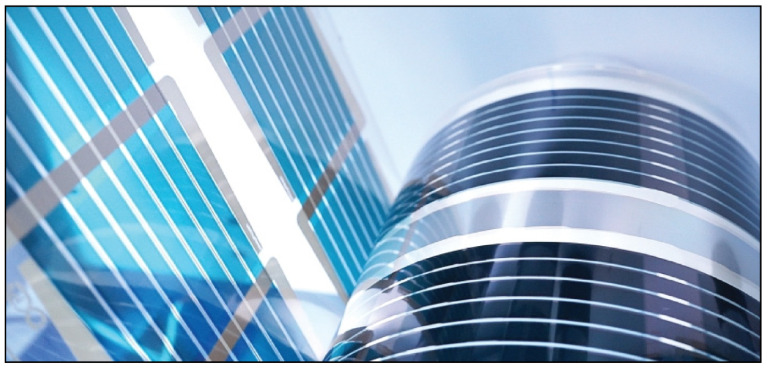
The capacity to produce the modules as big rolls of thin film using high-speed techniques is a main component of the low cost of developing photovoltaic devices.

**Figure 35:**
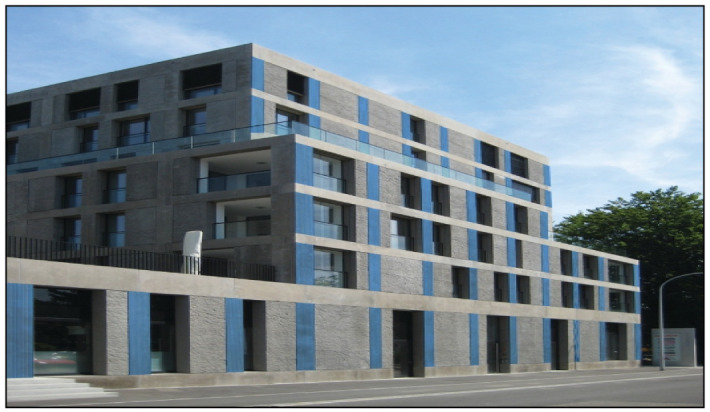
Thin organic solar-cell panels are not restricted to be used in merely glass on building facades but may also be designed to be integrated into cement and metal components.

However, a variety of cheap materials, including organic polymers, small molecules, and numerous inorganic substances, are employed to create the new OSCs. Moreover, unlike silicon cells, the developing ones can be produced and commercialized on flexible supports using inexpensive solution-phase processes that are utilized in the manufacture of organic polymers, such as high-speed roll-to-roll printing as shown in Figure 34 [147]. Figure 35 exhibited that thin organic solar-cell panels can be inserted into cement and metal pieces of a building façade in additional to glass.

The innovation of non-fullerene acceptors (NFAs) has allowed OSCs devices to reach greater efficiency. It was noted that OSC efficiency was considerably improved by using NFAs. The efficiency of OSCs has delivered an impressive PCE of over 18% in just a few short years. In comparison, OSCs are now at the lower end of the typical silicon solar cell’s efficiency range of 18 to 22 percent. Many specialists, some of whom started working in the subject when OSC efficiency tended to stay at only 3%, have stated that this increase in efficiency has surpassed their expectations [148].

**Figure 36:**
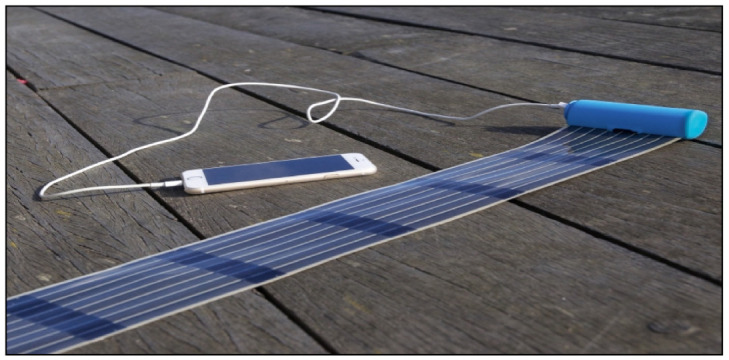
Ultra-thin layers and flexible organic photovoltaic panel for a phone-charging device.

In the African continent, millions of people are still using candles and kerosene lamps to light their houses, and open fires made of wood are being used to cook daily meals and heat water. However, this is hazardous, costly, risky, and polluting. Radios are frequently powered by batteries, and individuals frequently have to travel long distances only to charge their cell phones [149]. A flexible organic photovoltaic panel as depicted in Figure 36, which can be rolled up for storage, is essential for charging mobile devices.

Much more work must be achieved before OSCs can be extensively commercialized. One of the main drawbacks associated with OSC production is the selected solvents. Chlorinated solvents are commonly used in OSC manufacturing but these solvents can pose risks to human health and the environment. Professor Bernard Kippelen, at the Georgia Tech Institute of Electrical and Computer Engineering, said that “when scaling up OSC manufacturing, you have to consider the exposure of people who will be working in the manufacturing plants.” The research to date has essentially concentrated on achieving progressively enhanced efficiency, but as Kippelen says, “we need an approach that goes well beyond just one number.” The manufacturing approach needs to be improved to be safer and more economical for OSCs to be a commercially viable technology in the global markets [142]. Wu et al. have exhibited a list of OSC applications. For example, the two exemplary applications of flexible OSCs are biomedical devices and wearable electronics. When it comes to semitransparent OSC devices, smart windows and greenhouse uses are highly competitive. The Internet of Things and energy harvesting are two areas where indoor OSCs have many potential applications [150].

## 10. Summary and Perspectives

Numerous conjugated polymers have been intensively investigated and applied for a variety of optoelectronic devices, including PSCs, OLEDs, and OFETs. Conjugated polymers have gained significant interest due to their promise for low-cost, simple manufacturing and their application in light-emitting devices such as digital cameras and mobile phones are some of the kinds of flat display panels that are already commercialized. OFETs are critical elements in many industrial applications, including modern circuitry, single amplifiers, and electrical switches. To date, fossil fuels account for the majority of global energy use. Burning fossil fuels creates greenhouse gases such as CO_2_ that hurt the ecosystem and contribute to climate change, global warming, and air pollution. This has encouraged researchers to find renewable energy sources. A promising approach to meeting the world’s largest rising energy needs is to collect solar energy and use photovoltaic (PV) technology to turn it into electricity [151].

PSCs have received considerable attention as a renewable energy source because of their benefits including light weight, flexibility, and solution processing. The PCE of BHJ PSCs, in which the photoactive layer consists of a mixture of acceptor and donor, has significantly increased in the last decade [152]. The key to achieving the high photovoltaic performance of PSC is designing innovative conjugated polymers with a narrow energy band gap (*E_g_*) and proper energy level arrangement to absorb throughout a broad spectrum and have a good harvesting ability [153]. Over the years, researchers have developed D–A copolymers with high PCEs for organic photovoltaic (OPV) applications [154,155]. These polymers include fluorene, carbazole, anthracene, dibenzosilole (DBS), and cyclopentadithiophene (CPDT) for use as electron-donating monomers and 2,1,3-benzothiadiazole (BTDZ) and naphthothiadiazole (NT) for use as electron-accepting monomers [156]. The innovations in photovoltaic materials have contributed significantly to the PCE improvement of PSCs. Many novel materials have been invented for PSCs that exhibit 18% PCE. In addition, the constant advancements in donor and acceptor technology have brought ternary and tandem OSCs, which afforded PCEs exceeding 20%—a significant potential that competes with other solar technologies [157]. The control nanoscale morphology of the active layer in BHJ is another important parameter for achieving high-performance PSCs to secure efficient exciton dissociation at the D–A interface, the transport of electrons and holes in two separate phases, and their extraction at the contacts. Furthermore, the morphology of the active layer of devices has a noticeable influence on the *V_oc_*, *J_sc_*, and FF values. The nanomorphology of the photoactive layer can be affected by several processing parameters such as the choice of casting solvent, thermal and solvent annealing, additives, blend composition, and deposition method.

## Data Availability

Not applicable.
